# Identifying a novel cuproptosis-related necroptosis gene subtype-related signature for predicting the prognosis, tumor microenvironment, and immunotherapy of hepatocellular carcinoma

**DOI:** 10.3389/fmolb.2023.1165243

**Published:** 2023-05-23

**Authors:** Yuanxin Shi, Peng Qiu, Kai Zhao, Xiangyu Li, Yunxiang Feng, Zhengdong Deng, Jianming Wang

**Affiliations:** ^1^ Department of Biliary and Pancreatic Surgery, Cancer Research Center Affiliated Tongji Hospital, Tongji Medical College, Huazhong University of Science and Technology, Wuhan, China; ^2^ Department of Pediatric Surgery, Tongji Hospital of Tongji Medical College, Huazhong University of Science and Technology, Wuhan, China; ^3^ Affiliated Tianyou Hospital, Wuhan University of Science and Technology, Wuhan, China

**Keywords:** hepatocellular carcinoma, cuproptosis, necroptosis, prognostic signature, tumor microenvironment, immunotherapy

## Abstract

**Background**: Cuproptosis and necroptosis represent two distinct programmed cell death modalities implicated in neoplastic progression; however, the role of combining cuproptosis and necroptosis in hepatocellular carcinoma (HCC) remains to be elucidated.

**Methods**: A total of 29 cuproptosis-related necroptosis genes (CRNGs) were identified, followed by an extensive analysis of their mutational characteristics, expression patterns, prognostic implications, and associations with the tumor microenvironment (TME). Subsequently, a CRNG subtype-related signature was developed, and its value of prognostic prediction, TME, and therapeutic responses in HCC were thoroughly investigated. Last, quantitative real-time PCR and Western blotting were employed for investigating the signature gene expression in 15 paired clinical tissue samples.

**Results**: Two distinct CRNG subtypes were discerned, demonstrating associations between CRNG expression patterns, clinicopathological attributes, prognosis, and the TME. A CRNG subtype-related prognostic signature, subjected to external validation, was constructed, serving as an independent prognostic factor for HCC patients, indicating poor prognosis for high-risk individuals. Concurrently, the signature’s correlations with an immune-suppressive TME, mutational features, stemness properties, immune checkpoint genes, chemoresistance-associated genes, and drug sensitivity were observed, signifying its utility in predicting treatment responses. Subsequently, highly accurate and clinically convenient nomograms were developed, and the signature genes were validated via quantitative real-time PCR and Western blotting, further substantiating the stability and dependability of the CRNG subtype-related prognostic signature.

**Conclusion**: Overall, this investigation presented an extensive panorama of CRNGs and developed the CRNG subtype-related prognostic signature, which holds potential for implementation in personalized treatment strategies and prognostic forecasting for HCC patients.

## Introduction

Hepatocellular carcinoma (HCC), representing 80% of primary liver cancer cases, accounts for a third of cancer-related fatalities (Yang et al., 2019; Vogel et al., 2022). Surgical intervention remains the optimal therapeutic strategy for HCC; however, high recurrence rates persist as a challenge. Up to 80% of HCC patients experience recurrence within 5 years post-radical resection, and liver transplantation recurrence rates are approximately 15%. Unfortunately, only a few patients qualify for liver transplantation (Tabrizian et al., 2015; Vogel et al., 2022). Despite the fact that systemic therapies are favored for advanced or unresectable HCC, the treatment effects and options are limited. Sorafenib can extend the median survival of patients by 2–3 months, and lenvatinib demonstrates equal or superior potency (Llovet et al., 2008; Kudo et al., 2018). Nevertheless, most agents fail to improve patient survival with regorafenib being a rare exception (Bruix et al., 2017). Immunotherapy emerges as a promising HCC treatment option. Combined use of bevacizumab and atezolizumab results in improved overall survival (OS) compared to sorafenib, which is the sole approved immune checkpoint therapy for HCC. However, immunotherapy cannot consistently implement the desired effect. Consequently, delving into the heterogeneity and regulatory mechanisms among HCC patients, accurately assessing their clinical prognosis and enhancing the therapeutic outcomes of HCC are the current foci in the field of HCC research. The identification of novel biomarkers serves as a crucial approach to achieve these objectives and has been extensively employed in the stratification, prognostic evaluation, and treatment guidance of HCC patients. Cuproptosis, a novel programmed cell death (PCD) pathway linked to copper homeostasis, transpires through the following processes: intracellular copper directly binds to lipoylated components in the tricarboxylic acid cycle, thus inducing lipoylated DLAT oligomerization and iron–sulfur (Fe/S) cluster loss. Subsequently, proteotoxic stress activation triggers HSP70 induction, resulting in cell death (Tsvetkov et al., 2022). In recent years, some research studies have revealed associations between copper homeostasis and cancer development, suggesting that elevated copper levels promote malignant cancer behaviors and phenotypes, also called “cuproplasia” (Babak and Ahn, 2021; Ge et al., 2022). In HCC, the upregulated intracellular copper has been reported to confer radiotherapy resistance and contribute to malignant transformation in Wilson disease (Gunjan et al., 2017; Yang M. et al., 2022). Given cancer’s copper dependence, targeting cuproptosis may serve as a potential therapeutic strategy (Ge et al., 2022). Indeed, copper-based nanocarriers have demonstrated practical prospects for HCC treatment, potentially related to cuproptosis (Cai et al., 2021). Necroptosis shares similarities with cell apoptosis and necrosis (Vandenabeele et al., 2010; Oberst et al., 2011; Kaczmarek et al., 2013). Morphologically and immunologically, necroptosis manifests as membrane rupture, cytoplasmic concentration, organelle swelling, and robust immune response induced by damage-associated molecular pattern (DAMP) release. Molecularly, necroptosis is initiated by intracellular and extracellular stimuli akin to apoptosis, with pathway switching contingent upon caspase-8 status (caspase-8 inhibition activates necroptosis). Necroptosis exerts a dual influence on hepatocellular carcinoma progression. Some data of mice indicate necroptosis is associated with HCC risk factors, including chronic liver inflammation and fibrosis (Mohammed et al., 2021; Thadathil et al., 2022). On the contrary, downregulated RKIPs have been observed to foster HCC progression via immunosuppression and chemoresistance induction (Wu et al., 2020; Nicolè et al., 2022). Targeting necroptosis may also constitute an alternative HCC treatment, with preliminary evidence supporting this notion (Lan et al., 2020). Although the regulatory mechanisms of cuproptosis and necroptosis have been investigated independently, and several biomarkers based on cuproptosis or necroptosis-associated genes have been established as prognostic and therapeutic predictors, few studies have systematically examined the combined effects of these two cell death modalities in HCC. Moreover, it remains unknown whether the concomitant application of cuproptosis-related genes (CRGs) and necroptosis-related genes (NRGs) can serve as predictive factors for patient outcomes and treatment efficacy. Consequently, we identified 29 cuproptosis-associated necroptosis genes (CRNGs) by integrating CRGs and NRGs, performing an extensive analysis of their prognostic value, tumor microenvironment (TME) associations, and treatment responses in HCC with the aim of elucidating the collaborative roles of these two programmed cell death pathways in this malignancy.

## Materials and methods

### Collection and processing of data

The gene expression profiles and clinical information of three independent HCC cohorts (TCGA-LIHC, ICGC-LIRI-JP, and GSE14520) were acquired from The Cancer Genome Atlas (TCGA) (https://portal.gdc.cancer.gov/), the International Cancer Genomics Consortium (ICGC) (https://icgc.org/), and Gene Expression Omnibus (GEO) (https://www.ncbi.nlm.nih.gov/geo/). For all the RNA-seq data, transformation of fragments per kilobase million (FPKM) values into transcripts per kilobase million (TPM) values has been conducted using the “limma” package. Batch effect elimination between different cohorts was performed using the “SVA” package, and all data underwent log2 transformation. In total, 365 patients of TCGA-LIHC with gene expression profiles, somatic mutation information, copy number variation (CNV), and clinicopathological information were utilized as an entire data cohort for the subsequent analyses. In addition, 231 ICGC-LIRI-JP patients and 200 GSE14520 patients served as external validation cohorts.

The cuproptosis-related genes (Supplementary Table S1) and necroptosis-related genes (Supplementary Table S2) were sourced from previous studies (Zhao et al., 2021; Peng X. et al., 2022) and the GeneCards website (https://www.genecards.org/).

### Identification of cuproptosis-related necroptosis genes and unsupervised clustering analysis

In total, 29 CRNGs were identified by the correlation analysis of CRG and NRG expression in HCC patients, adhering to the following criteria: |R|>0.6 and *p*-value <0.001 (Supplementary Tables S3-S4). Consensus unsupervised clustering analysis was conducted by the “Consensus Cluster Plus” package based on CRNG expressions, and principal component analysis (PCA) was applied for showing the differences in CRNG subtypes. Subsequently, R packages “survival” and “survminer” were employed for survival analysis, and differences in clinicopathological features were visualized using the “pheatmap” package. Meanwhile, immune checkpoint genes (ICGs) and chemoresistance-related genes (CRRGs) were extracted from several previous research studies (Marin et al., 2020; Hu et al., 2021) and the GeneCards website. Differential expression patterns of these genes were compared among HCC patients. In addition, single-sample gene set enrichment analysis (ssGSEA) was used for estimating immune cell infiltration levels.

### Analysis of functional enrichment

To compare the biological functions and signaling pathways in patients with different CRNG subtypes, Gene Set Variation Analysis (GSVA) was conducted with terms such as “h.all.v7.2. symbols” and “c2. cp.kegg.v7.5. symbols” via the “GSVA” package. Gene Set Enrichment Analysis (GSEA) was performed with terms including “h.all.v7.2. symbols” and “c2. cp.kegg.v7.5. symbols” following the criteria *p*-value <0.05, FDR <0.25, and NES >1. Subsequently, the “limma” package was applied for identifying differentially expressed genes (DEGs) between CRNG subtypes, adhering to the criteria |lg FC| > 0.585 and *p*-value <0.05. Gene Ontology (GO) and Kyoto Encyclopedia of Genes and Genomes (KEGG) enrichment analyses were conducted using the R “clusterProfiler” package then.

### Identification of differentially expressed genes related to overall survival and consensus unsupervised clustering analysis

Of all CRNG subtype-related DEGs, over-survival-related DEGs (OS-related DEGs) were identified via Cox regression analysis with univariate variables. Consensus unsupervised clustering analysis and PCA analysis were carried out as previously described. A comparison of overall survival and clinicopathological features between gene clusters was then performed.

### Construction and evaluation of the prognostic signature related to the cuproptosis-related necroptosis gene subtype

Here, a novel cuproptosis-related necroptosis gene subtype-related signature was developed by Lasso Cox regression analysis. Patients of the TCGA-LIHC cohort were randomly allocated to two cohorts (test and training cohorts at a ratio of 2:3) using the “caret” package first. The Lasso Cox regression analysis was performed using the “glmnet” package, and the penalty parameter lambda was selected based on the minimum criteria through 10-fold cross validation. Key genes of the CRNG subtype-related prognostic signature were determined through Cox regression analysis with multivariate variables, and GSEA analysis was performed immediately afterward. Next, risk scores were calculated for each HCC patient following the formula $risk\;score=\sum_{i=1}^{\infty }\left(Coefi\times Expi\right)$ (Coefi representing the coefficient and Expi representing key gene expressions). Patients were classified as high or low risk by comparing their own risk scores to the median risk score. To evaluate the predictive value of the prognostic signature, survival analysis was performed using the “survival” and “suvminer” packages. Receiver operating characteristic (ROC) analysis was conducted by the R “timeROC” package, and signature gene expression levels as well as OS in patients of the two groups were visualized by the heatmap and scatter chart. In addition, ICGC-LIRI-JP and GSE14520 cohorts were utilized for external validation of the CRNG subtype-related prognostic signature with the same analyses performed as described previously.

### Analysis of independent prognostic factors and construction of clinical nomograms

Here, Cox regression analysis with univariate, multivariable variables, and concordance index (C-index) analysis were performed to identify independent prognostic factors. Based on the CRNG subtype-related signature and clinical characteristics, clinical nomograms were constructed through the “rms” and “regplot” packages for patients in three cohorts, respectively. Curves of calibration and ROC were generated for evaluating the accuracy of the clinical nomograms.

### Correlation analysis of the prognostic signature, immune status, and stemness characteristics in HCC patients

GSEA analysis was executed to identify significant discrepancies in biological functions and signaling pathways between high-risk and low-risk patients. The immune cells’ infiltration levels in patients of the TCGA-LIHC cohort were assessed utilizing the CIBERSORT algorithm. Subsequently, correlation analyses between this signature and immune cell infiltration levels were performed, as was an evaluation of the tumor microenvironment scores using the “ESTIMATE” package. In addition, a broader landscape of immune cell infiltration was explored through multiple methods, such as CIBERSORT-ABS, TIMER, and XCELL. Concerning immune functionality, disparities between the two patient groups were determined by employing the R package “GSVA” and the single-sample GSEA (ssGSEA) algorithm. Last, tumor stem cell attributes of patients in the TCGA-LIHC cohort were extracted from “StemnessScores_RNAexp_20170127.2. tsv,” and a correlation analysis on stemness characteristics and risk scores was conducted.

### Correlation analysis of the prognostic signature, immune checkpoint genes, and tumor mutation burden

The “maftools” package was applied for displaying gene mutational features in patients with different risk profiles. Next, the tumor mutation burden (TMB) score was calculated, and a correlation analysis was conducted, involving the TMB score, prognostic signature, gene clusters, and overall survival. In addition, a correlation analysis between the prognostic signature and ICGs was conducted.

### Validation of the prognostic signature in immunotherapy response

The Tumor Immune Dysfunction and Exclusion (TIDE) algorithm (http://tide.dfci.harvard.edu/) was employed for comparing immunotherapy response between patients with different risk profiles. Another four cohorts, GSE120644, GSE78220, checkmate, and IMvigor210, were employed for further corroborating the predictive capacity of the prognostic signature in determining immunotherapy outcomes.

### Correlation analysis of the prognostic signature, chemoresistance-related genes, and drug sensitivity

A comprehensive correlation analysis was performed to examine the interplay between chemoresistance-associated gene expression profiles and prognostic signatures. The “oncoPredict” package was employed for estimating the differential sensitivity to prevalent hepatocellular carcinoma chemotherapeutic agents among patients with distinct risk stratifications.

### Clinical tissue sample collection, quantitative real-time PCR, and Western blot analysis

A total of 15 matched hepatocellular carcinoma tissue specimens and para-tumor tissues were procured from the Affiliated Tongji Hospital, Huazhong University of Science and Technology, China. Ethical approval was granted by the Tongji Hospital Research Ethics Committee. The clinicopathological information of patients is presented in Supplementary Table S5. Total RNA extraction from clinical tissue samples was extracted using TRIzol™ reagent (Invitrogen, Catalog #15596026), followed by complementary DNA synthesis using PrimeScript™ RT Master Mix (Takara Bio Inc., Catalog #RR036A) in accordance with the manufacturer’s guidelines. Quantitative real-time PCR (qRT-PCR) was executed employing a SYBR Premix EX Taq kit (Takara Bio Inc., Catalog #RR420A), adhering to the prescribed protocol. The results were analyzed as previously delineated (Yang T. et al., 2022). Western blotting (WB) was carried out in accordance with previously established methodologies (Dobin et al., 2013). Image Lab software facilitated the quantification of specific band intensities. The primers and antibodies involved here are detailed in Supplementary Table S6.

## Statistical analysis

Statistical computations were performed using R software (v.4.1.3) and GraphPad Prism (v.8.0.1). GSEA analysis was completed using GSEA software (v.4.2.3). Quantitative data are expressed as mean ± standard deviation (SD). Independent Student’s *t*-test was used to determine the significance of differences between two groups. Spearman’s correlation test was applied for correlation analysis. Survival analysis was conducted using the Kaplan–Meier method. The “maftools” package facilitated the establishment of the variation features of CRNGs. Segmentation analysis, the GISTIC algorithm, and the “RCircos” package were utilized for the comprehensive analysis of CNV. *p*-value <0.05 indicated statistical significance.

## Results

### Mutation and expression features of CRNGs in HCC

**FIGURE 1:**
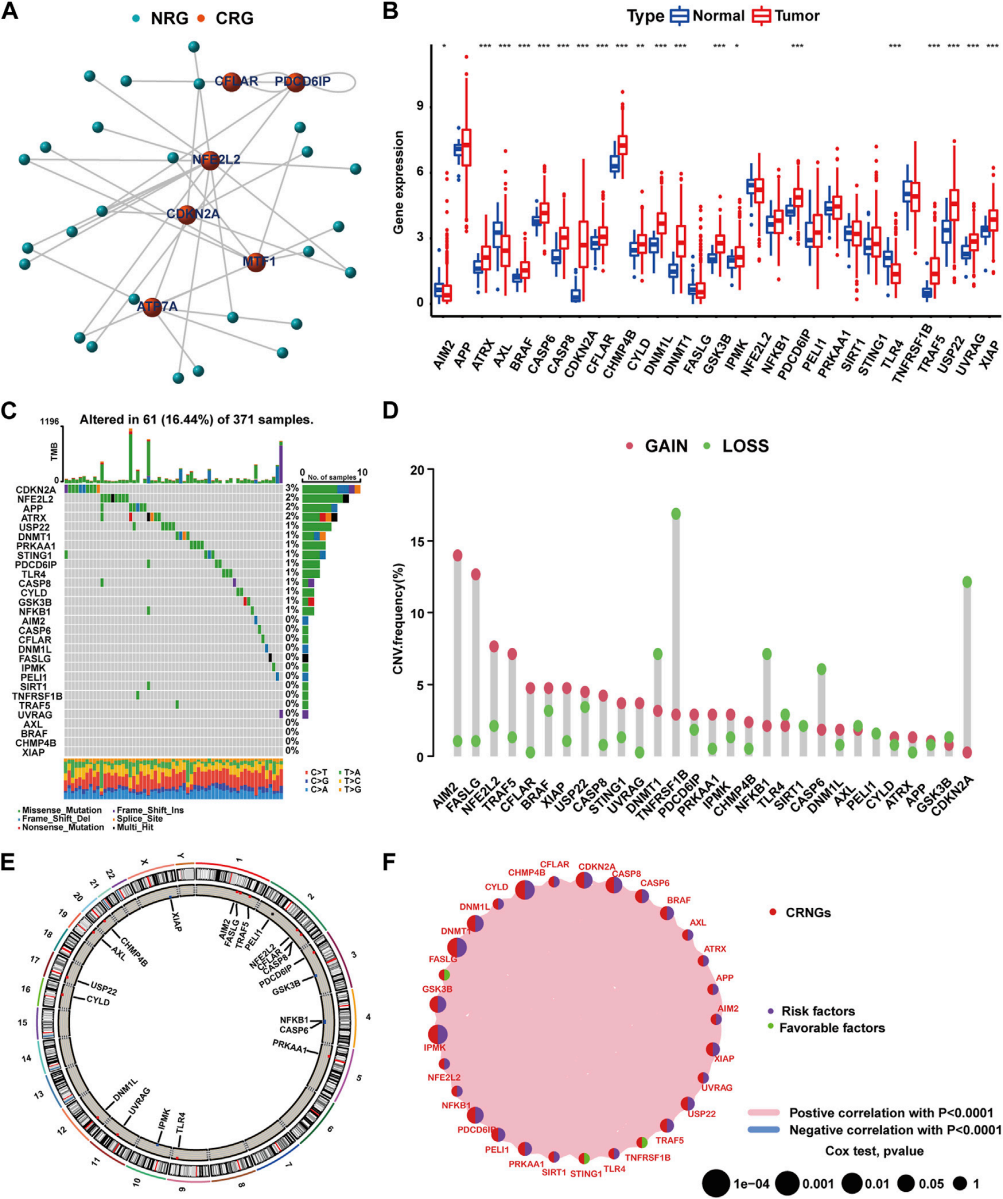
Mutation and expression features of CRNGs in HCC. **(A)** Identification of CRNGs based on the correlations between the expression of CRGs and NRGs. **(B)** Differential expression of 29 CRNGs in HCC. **(C)** Landscape of CRNGs mutations in HCC patients. **(D)** CNV analysis on CRNGs of HCC patients. **(E)** Chromosomal location of CNV alteration in CRNGs. **(F)** Interactions among CRNGs in HCC (*p-value <0.05, **p-value <0.01, and ***p-value <0.001).

The flowchart of this work is delineated in Supplementary Figure S1. Through Spearman’s correlation analysis, 29 CRNGs were identified, and their interaction network is shown in Figure 1A. The differential expression of CRNGs is shown in Figure 1B, wherein 17 CRNGs were significantly upregulated in tumor tissues, and two CRNGs exhibited significant downregulation. The gene mutation landscape of 29 CRNGs is shown in Figure 1C with 61 patients (16.44%) in the TCGA-LIHC cohort exhibiting CRNG mutations, and CDKN2A and NFE2L2 emerged as the most frequently mutated genes. CNV frequency is shown in Figure 1D, where AIM2, FASLG, NFE2L2, TRAF5, and CFLAR demonstrated elevated frequencies, while TNFRSF1B, CDKN2A, DNMT1, NFKB1, and CASP6 exhibited higher loss frequencies; Figure 1E establishes the alterations and chromosomal locations of CNV in 29 CRNGs. The interaction network of the 29 CRNGs is shown in Figure 1F. In addition, 15 CRNGs related to prognosis were identified via survival analysis employing the Kaplan–Meier method and univariate Cox regression (Supplementary Figure S2, Supplementary Table S7). Further analysis revealed CHMP4B as an independent prognostic factor for HCC patients using multivariate Cox regression, as indicated in Supplementary Table S8.

### Correlations of CRNG subtypes with clinical features, CRRGs and ICGs, and immune cell infiltration

**FIGURE 2:**
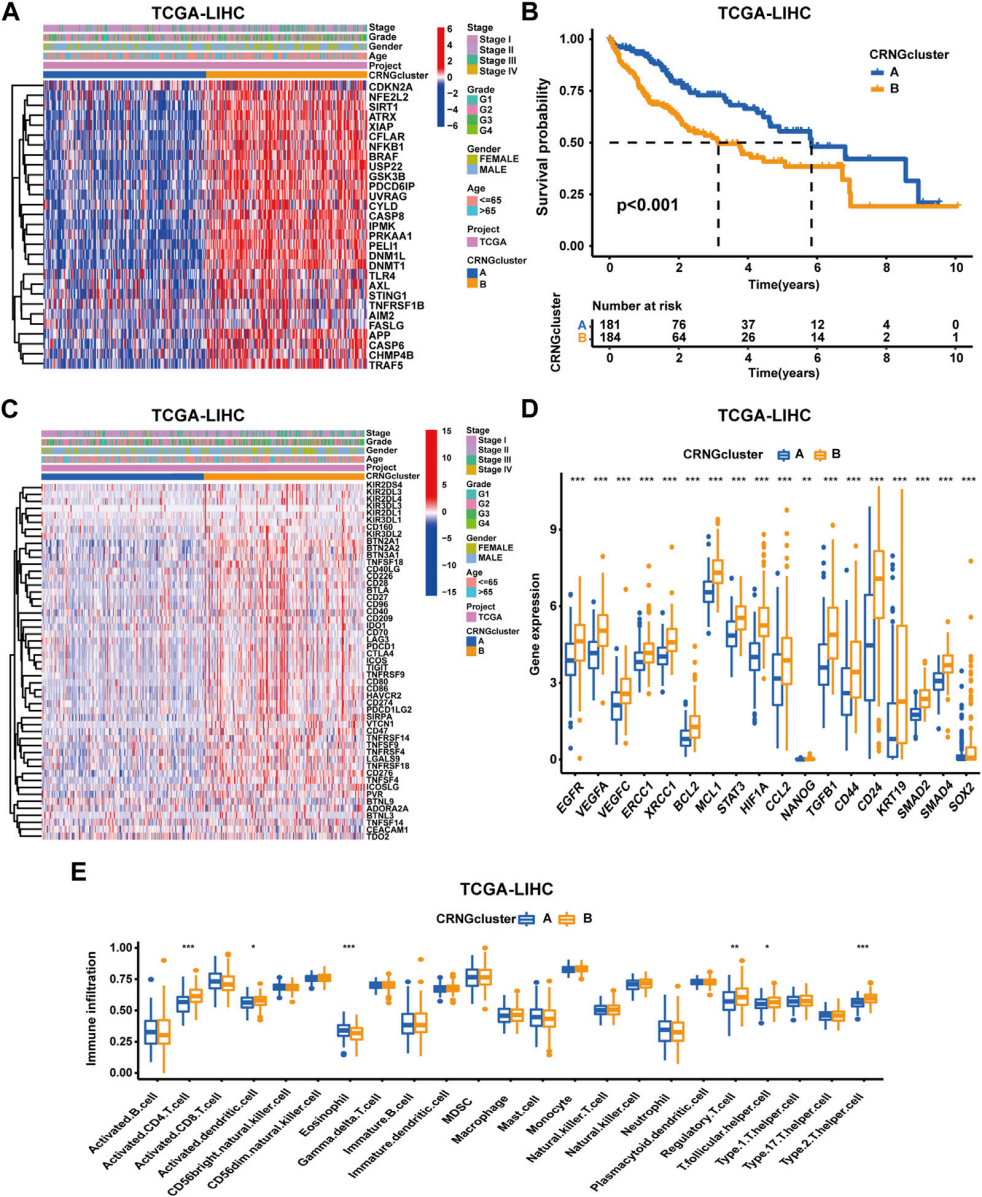
Correlations of CRNG subtypes with clinical features, CRRGs, ICGs, and immune cell infiltration. **(A)** Different CRNG expression patterns and clinicopathological features between two CRNG subtypes. **(B)** Survival analysis on patients of two subtypes. **(C)** Different ICG expression patterns and clinicopathological features in patients of two subtypes. **(D)** Different CRRG expression patterns between patients of two subtypes. **(E)** Different immune cell infiltration patterns between patients of two subtypes (*p-value <0.05, **p-value <0.01, and ***p-value <0.001).

According to 29 CRNGs expression levels, consensus clustering analysis was executed, thus resulting in the classification of patients into CRNG subtype A (subtype A = cluster A) and CRNG subtype B (subtype B = cluster B) (Supplementary Figure S3A-H). Principal component analysis unveiled a marked distinction between the two CRNG subtypes (Supplementary Figure S3I). Subsequently, an exhaustive correlation analysis was undertaken. Divergent CRNG expression patterns and clinicopathological features in the two CRNG subtypes are shown in Figure 2A, indicating that CRNG subtype B is associated with elevated CRNG expression levels and advanced clinical staging. The survival curves revealed that patients of CRNG subtype B experienced inferior overall survival compared to those of CRNG subtype A (Figure 2B). These findings implied that CRNG subtype B was associated with a detrimental prognosis. Meanwhile, patients of CRNG subtype B exhibited augmented expression levels of ICGs and CRRGs (Figures 2C,D). In addition, distinct immune cell infiltration levels were observed between the two subtypes. Through “ssGSEA” algorithms, it was determined that CRNG subtype B correlated with increased infiltration levels of five immune cells, while only eosinophils demonstrated enhanced infiltration in CRNG subtype A (Figure 2E).

### Comparative analysis of functional enrichment in patients with two CRNG subtypes

**FIGURE 3:**
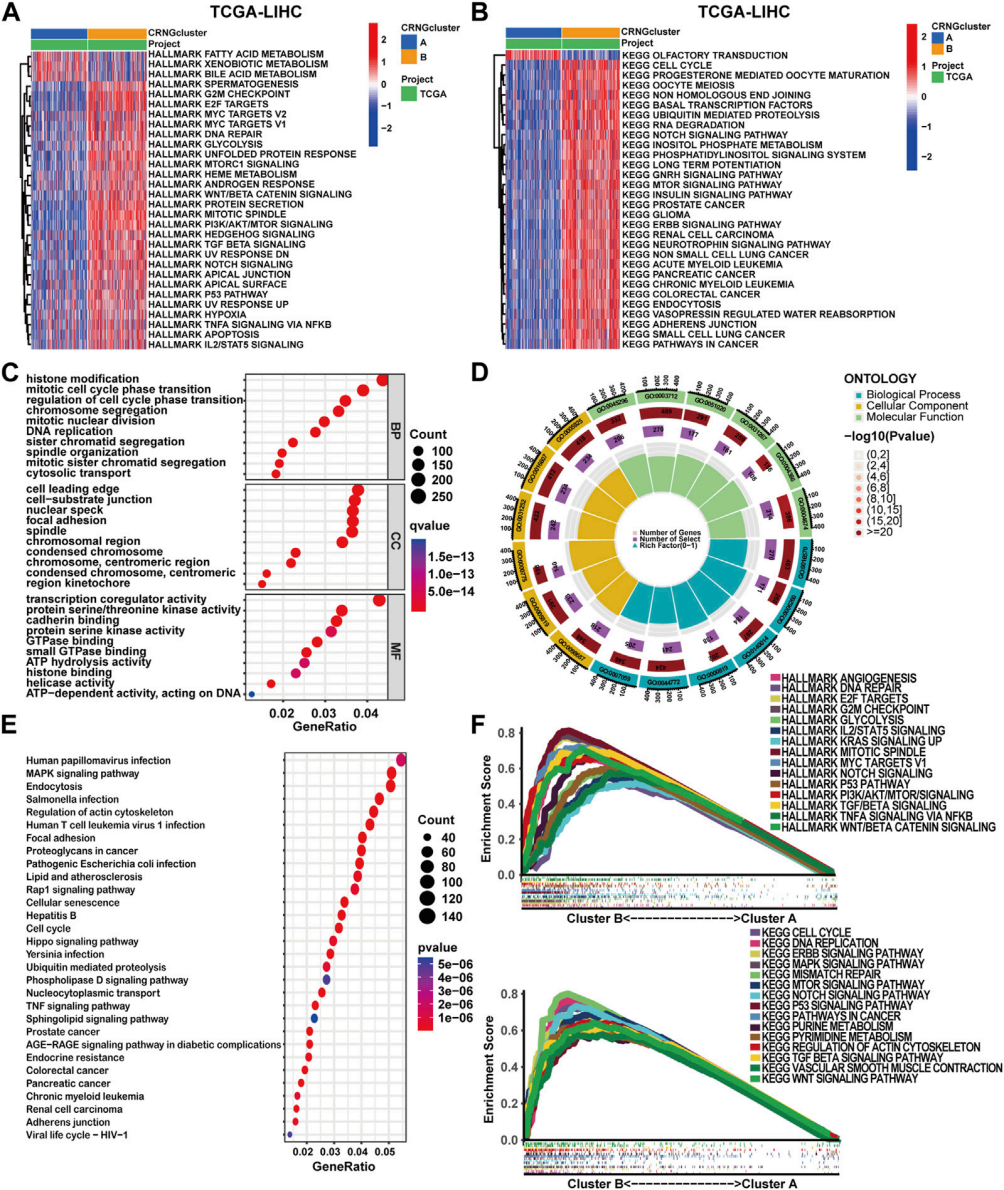
Comparative analysis of functional enrichment in patients with two CRNG subtypes. **(A)** GSVA analysis with HALLMARK terms between patients of two subtypes. **(B)** GSVA analysis with KEGG terms between patients of two subtypes. **(C–E)** Analysis of GO and KEGG enrichment on DEGs between patients of two subtypes. **(F)** GSEA analysis on DEGs between patients of two subtypes.

To delineate the distinct biological function characteristics between patients of CRNG subtype A and B, GSVA, GSEA, and CO/KEGG analyses were conducted. Both GSVA and GSEA analyses revealed a significant enrichment of biological processes and signaling pathways implicated in cuproptosis, necroptosis, and oncogenesis within patients of CRNG subtype B (Figures 3A, B, F) (Supplementary Tables S9–S10). Furthermore, 7,028 differential genes between CRNG subtypes were identified and GO/KEGG analyses were performed. As a result, an enrichment of biological processes associated with malignant cancer phenotypes or immunological responses was observed, including cell cycle, epithelial-to-mesenchymal transition (EMT), angiogenesis, checkpoint pathway, and Th1/Th2 cell differentiation. The enrichment of processes associated with cuproptosis and necroptosis, such as protein polymerization, protein degradation, protein acylation, TNF, and TLR signaling pathways was also observed (Figures 3C–E) (Supplementary Table S11).

### Identification of the gene subtype in HCC patients

**FIGURE 4:**
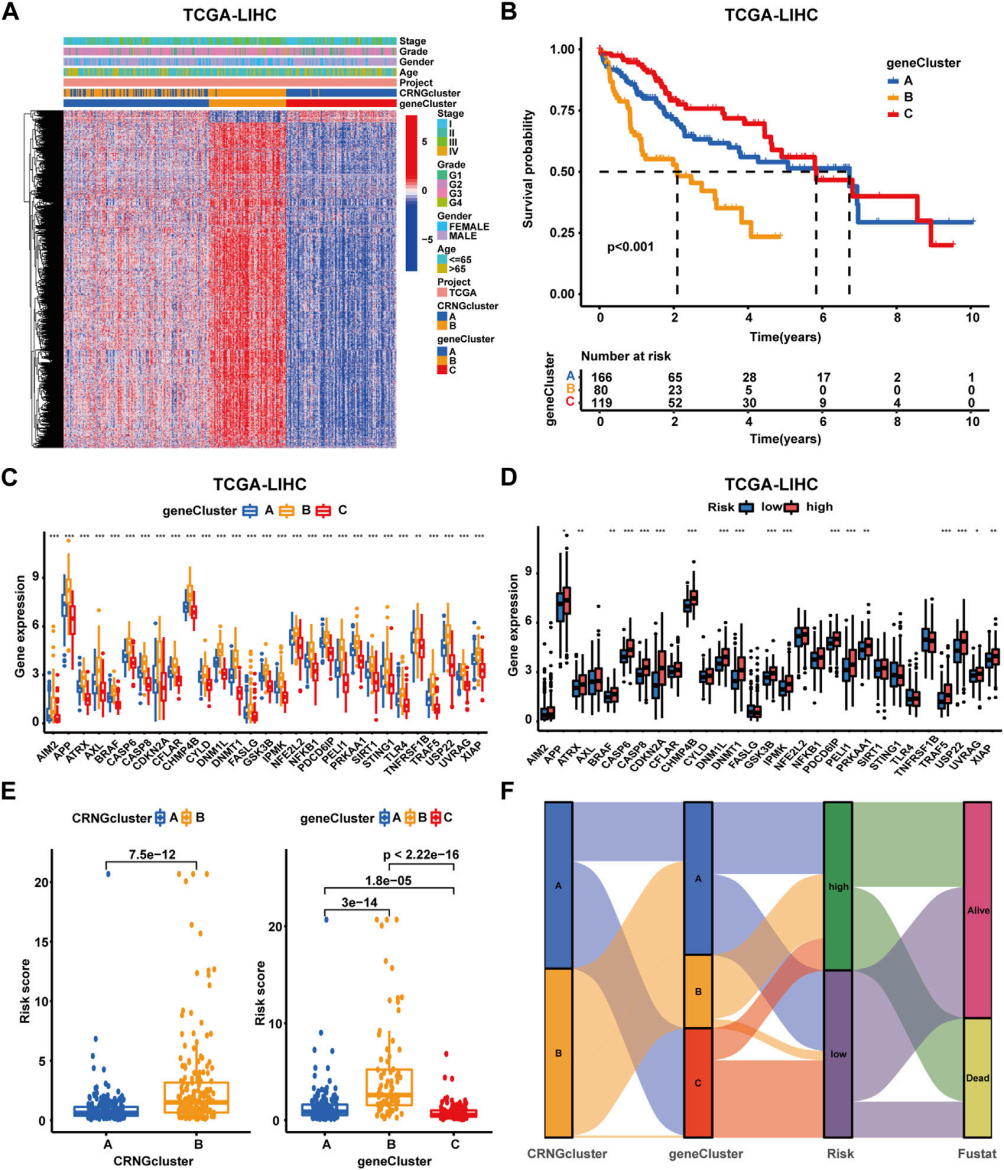
Identification and construction of gene subtypes and the CRNG subtype-related prognostic signature in HCC patients. **(A)** Correlation analysis on gene subtypes with OS-related DEGs, clinicopathological features, and CRNG subtypes. **(B)** Survival analysis on patients in three gene subtypes. **(C)** Different CRNG expression patterns among three gene subtypes. **(D)** CRNG expression patterns between patients with different risk profiles. **(E)** Correlation analyses on the risk score with CRNG subtypes and gene subtypes. **(F)** Correlation analyses of this prognostic signature with CRNG subtypes, gene subtypes, and survival status (*p-value <0.05, **p-value <0.01, and ***p-value <0.001).

Through univariate Cox regression analysis, 3,855 overall survival-related differentially expressed genes were identified. Subsequently, consensus clustering analysis was performed, categorizing patients into gene subtype A (subtype A = cluster A), gene subtype B (subtype B = cluster B), and gene subtype C (subtype C = cluster C) (Supplementary Figure S4A-H). PCA analysis demonstrated significant distinctions among these three molecular subtypes (Supplementary Figure S4I). A heatmap and survival curves were applied to compare OS-DEG expression levels, clinicopathological features, CRNG clusters, and OS among patients within the three clusters (Figures 4A, B), in addition to comparing CRNG expression (Figure 4C). Notably, patients within gene subtype B exhibited the poorest prognosis, while those within subtype C displayed the most favorable outcomes. Furthermore, CRNG expression progressively increased from gene subtypes C to A and B.

### Construction and validation of the CRNG subtype-related prognostic signature

**FIGURE 5:**
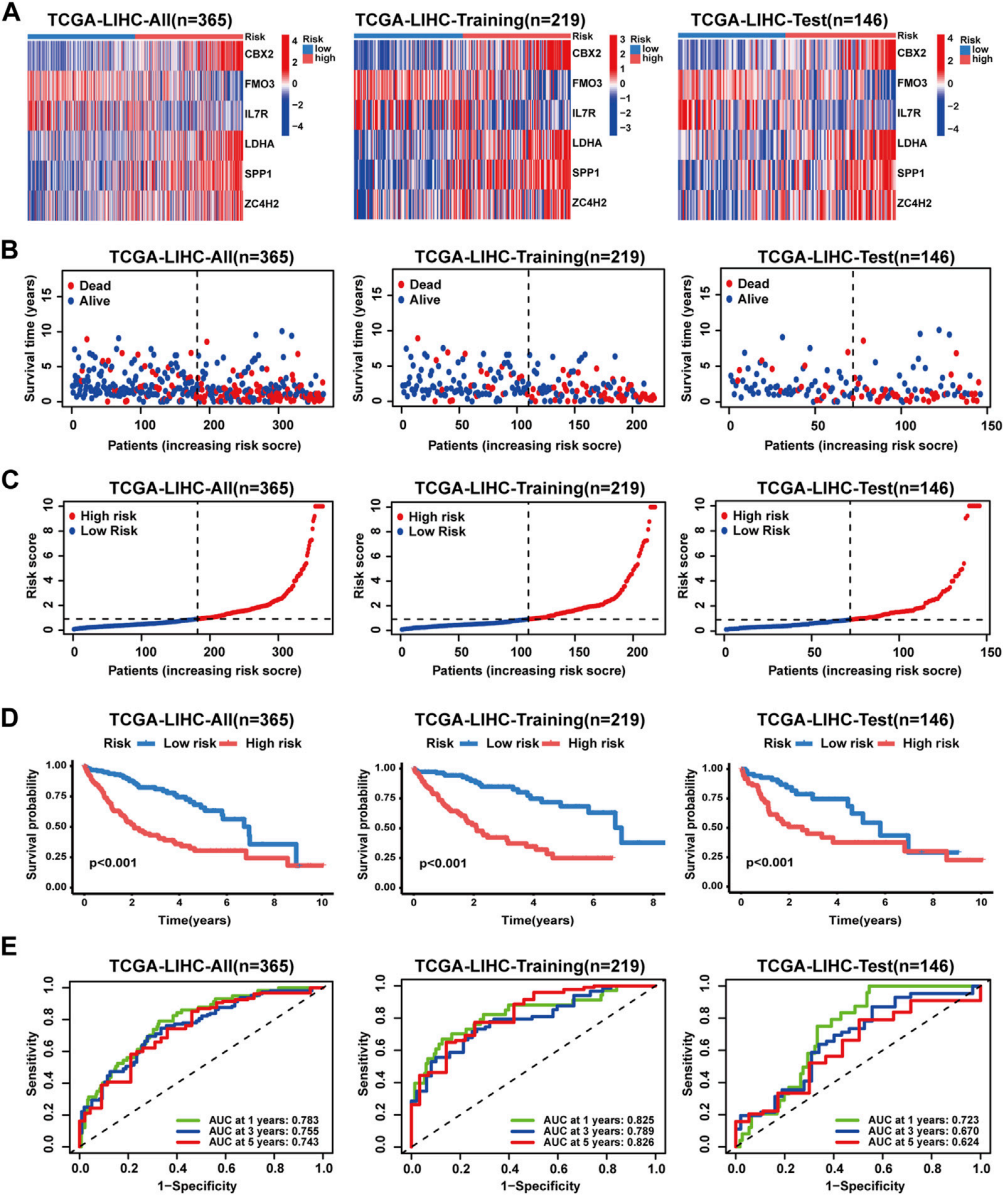
Validation of the prognostic signature in the TCGA-LIHC cohort. **(A)** Key gene expression patterns between patients with different risk profiles. **(B)** Distribution of survival time and status in patients with different risk profiles. **(C)** Distribution of the risk score in patients with different risk profiles. **(D)** Survival analyses of patients with different risk profiles. **(E)** ROC analyses of patients with different risk profiles.

In the TCGA-LIHC cohort, patients were categorized as test and training cohorts randomly at a 2:3 ratio (Table 1). A CRNG subtype-related prognostic signature was constructed through Lasso and multivariate Cox analyses. This signature encompassed six key genes, and GSEA analysis of key genes was performed, revealing the enrichment of biological processes associated with cancer initiation and progression, immune responses, and multiple metabolic pathways (Supplementary Figure S7) (Supplementary Table S12-17). The risk score was calculated as follows: risk score=CBX2*0.348049685−FMO3*0.13238182−IL7R*0.42489971+LDHA*0.42036947+SPP1*0.108648316+ZC4H2*0.388943111 (Table 2). Patients were identified as different risk profiles based on the comparison with the median risk score. Moreover, correlation analysis on the prognostic signature, CRNG subtypes, and gene subtypes was performed. We observed elevated CRNG expression levels in high-risk patients, with risk scores increasing from CRNG subtype A to B and from gene subtype C to A and B (Figures 4D, E). A Sankey diagram was employed to illustrate the distribution of patient subtypes across different risk levels and survival statuses (Figure 4F). Next, internal validation of the prognostic signature was performed. Heatmaps were utilized for visualizing key gene expression patterns, revealing the increased expression of CBX2, SPP1, LDHA, and ZC4H2 in patients at high risk, while IL-7R and FMO3 expression decreased in these patients (Figure 5A). Distribution plots of the prognostic signature demonstrated that the number of death cases rose with increasing risk scores, and survival curves depicted poorer OS in high-risk patients (*p*-value<0.001) (Figures 5B–D). ROC curves exhibited AUC values of 0.783, 0.755, and 0.743 for 1, 3, and 5 years, respectively, in the entire cohort, 0.825, 0.789, and 0.826 in the training cohort, and 0.723, 0.670, and 0.624 in the testing cohort (Figure 5E). Collectively, these results indicated a robust performance of this prognostic signature for HCC patients. By external validation, we further confirmed the prediction value. HCC patients were also divided into high- and low-risk groups, as previously described. In the ICGC-LIRI-JP cohort and GSE14520 cohorts (Supplementary Table S18), survival curves described poorer OS in high-risk patients. ROC curves displayed AUC values of 0.743, 0.743, and 0.886 for 1, 3, and 5 years, respectively, in the ICGC-LIRI-JP cohort and 0.673, 0.699, and 0.657 for 1, 3, and 5 years in the GSE14520 cohort (Supplementary Figure S8A-B) (Supplementary Figure S9A-B).

**TABLE 1:** Clinicopathological information of patients in the TCGA-LIHC cohorts.

Characteristics		Entire cohort		Training cohort		Test cohort		p-value
Age	<=65	227	62.19%	134	61.19%	93	63.70%	0.708
	>65	138	37.81%	85	38.81%	53	36.30%	
Gender	FEMALE	119	32.60%	74	33.79%	45	30.82%	0.6322
	MALE	246	67.40%	145	66.21%	101	69.18%	
Grade	G1	55	15.07%	34	15.53%	21	14.38%	0.8927
	G2	175	47.95%	105	47.95%	70	47.95%	
	G3	118	32.33%	72	32.88%	46	31.51%	
	G4	12	3.29%	6	2.74%	6	4.11%	
	unknow	5	1.37%	2	0.91%	3	2.05%	
Stage	Stage I	170	46.58%	100	45.66%	70	47.95%	0.979
	Stage II	84	23.01%	50	22.83%	34	23.29%	
	Stage III	83	22.74%	50	22.83%	33	22.60%	
	Stage IV	4	1.10%	2	0.91%	2	1.37%	
	unknow	24	6.58%	17	7.76%	7	4.79%	

**TABLE 2:** Key genes for the cuproptosis-related necroptosis gene subtype-related prognostic signature.

Key genes	Coefficient
*CBX2*	0.348049685
*FMO3*	−0.13238182
*IL7R*	−0.42489971
*LDHA*	0.42036947
*SPP1*	0.108648316
*ZC4H2*	0.388943111

### Independent prognostic analysis and construction of clinical nomograms

**FIGURE 6:**
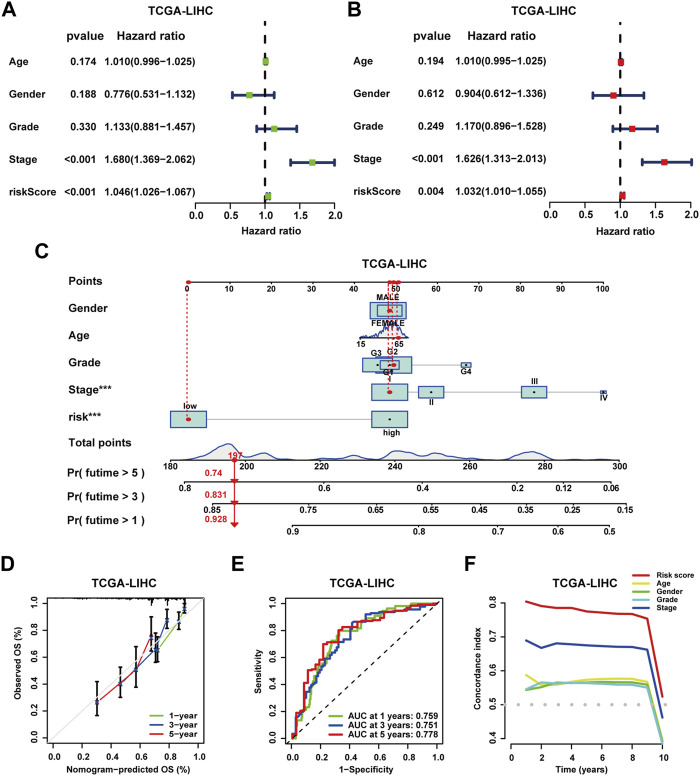
Independent prognostic analysis and construction of the clinical nomogram. **(A)** Analysis of Cox regression with univariate variables on the prognostic signature and clinical characteristics of patients in the TCGA-LIHC cohort. **(B)** Analysis of Cox regression with multivariate variables on the prognostic signature and clinical characteristics of patients in the TCGA-LIHC cohort. **(C)** Construction of clinical nomograms for predicting the OS probability of patients in the TCGA-LIHC cohort. **(D)** Assessment of the predictive value of clinical nomograms for patients in the TCGA-LIHC cohort with calibration curves. **(E)** Assessment of the predictive value of clinical nomograms for patients in the TCGA-LIHC cohort with ROC curves. **(F)** C-index analysis for independent prognostic value on the prognostic signature of patients in the TCGA-LIHC cohort.

To further evaluate the prognostic signature, analyses of Cox regression with univariate and multivariate variables were performed. In the TCGA-LIHC cohort, both the prognostic signature and clinical stage emerged as independent prognostic indicators (Figures 6A, B). In addition, the concordance index (C-index) curve exhibited superior performance in prognostic prediction of the signature (Figure 6F). In the ICGC-LIRI-JP and GSE14520 cohorts, the prognostic signature also served as an independent prognostic predictor, demonstrating optimal predictive performance (Supplementary Figure S8C-D, H) (Supplementary Figure S9C-D, H). Next, clinical nomograms were developed for patients in each of the three cohorts separately (Figure 6C, Supplementary Figure S8E, Supplementary Figure S9E). The calibration curves described an excellent consistency between predicted and observed outcomes (Figure 6D, Supplementary Figure S8F, Supplementary Figure S9F). ROC curves demonstrated the high accuracy of the nomograms for prognostic prediction. In TCGA-LIHC cohort patients, the AUC values were 0.759, 0.751, and 0.778 for 1, 3, and 5 years, respectively (Figure 6E); in the ICGC-LIRI-JP cohort, AUC values were 0.871, 0.754, and 0.824 for 1, 3, and 5 years (Supplementary Figure S8G); and in the GSE14520 cohort, AUC values were 0.749, 0.775, and 0.756 for 1, 3, and 5 years (Supplementary Figure S9G).

### Correlations of the prognostic signature with immune function, immune cell infiltration, and stemness characteristics in HCC

**FIGURE 7:**
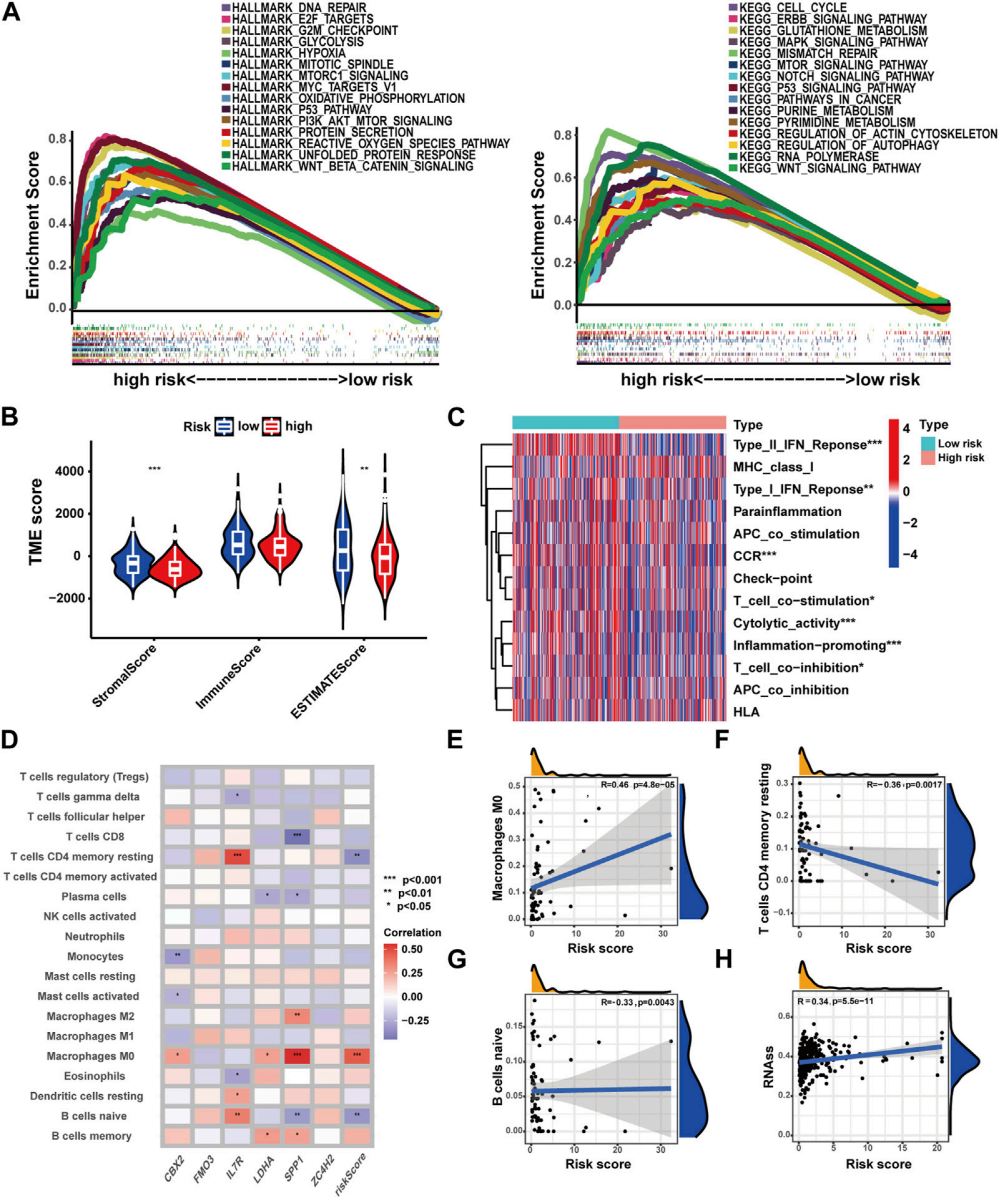
Correlations of the prognostic signature with immune function, immune cell infiltration, and stemness characteristics in HCC. **(A)** GSEA analysis with HALLMARK and KEGG terms of patients with different risk profiles. **(B)** TME score in patients with different risk profiles. **(C)** Immune function in patients with different risk profiles. **(D)** Correlations of immune cell infiltration with the prognostic signature. **(E–G)** Correlation analysis of immune cell infiltration and risk score. **(H)** Correlation analysis of the stemness score and risk score (*p-value <0.05, **p-value <0.01, and ***p-value <0.001).

To investigate biological function characteristics associated with different risk profiles, GSEA analysis was performed. The results revealed enrichment of cancer-related pathways and biological processes, including G2M checkpoint, E2F targets, cell cycle, genomic stability, and various metabolic pathways in high-risk patients (Figure 7A) (Supplementary Table S19). As shown in Figures 7B, C, lower stromal scores and estimate scores were observed in high-risk patients, while no significant difference in immune scores was detected here. In high-risk patients, most immune functions were suppressed, but checkpoint-related functions exhibited no significant differences between the groups. For a broader landscape, multiple algorithms were employed to compare immune cell infiltration levels in patients with different risk profiles (Figure 7D, Supplementary Figure S6). Based on the CIBERSORT algorithm, a positive correlation was observed between M0 macrophages and risk score, while naive B cells and CD4^+^ memory resting T cells displayed negative correlations with the risk score (Figures 7E–G). In addition, a positive association was identified between risk score and stemness score, which could lead the poor clinical outcomes in high-risk patients (Figure 7H).

### Correlations of the prognostic signature with the gene mutation landscape and ICGs in HCC

**FIGURE 8:**
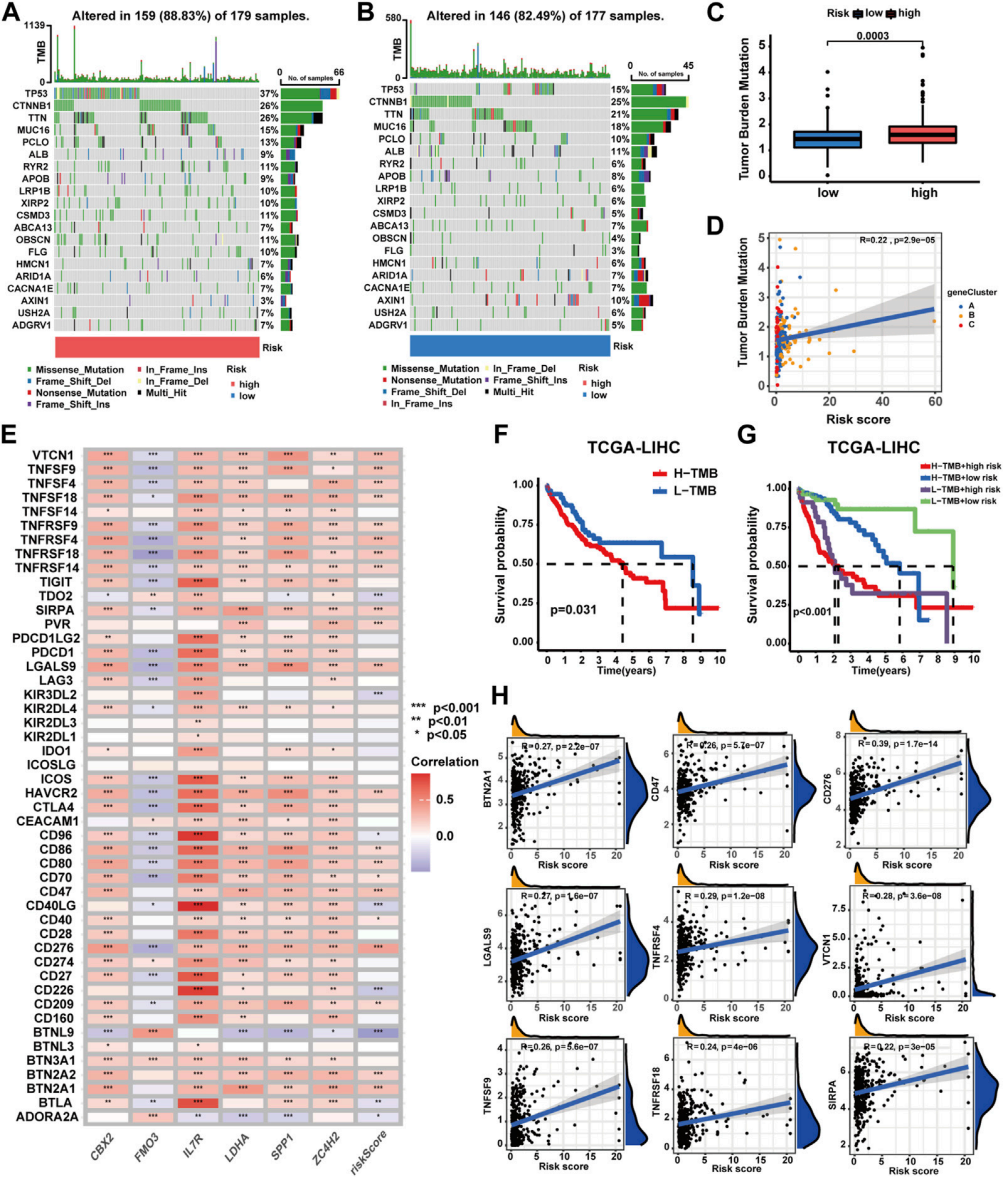
Correlations of the prognostic signature with the landscape of gene mutation and ICGs in HCC. **(A**–**B)** Gene mutation patterns between patients with different risk profiles. **(C)** Correlation analyses of the TMB score, prognostic signature, and gene subtypes. **(D)** TMB score between patients with different risk profiles. **(E)** Correlation analyses of the prognostic signature and ICG expression levels. **(F)** Survival analysis on patients with different TMB profiles. **(G)** Survival analyses on patients in four groups categorized by the TMB score and risk score. **(H)** Correlation analysis on ICG expression levels and the risk score (*p-value <0.05, **p-value <0.01, and ***p-value <0.001).

As shown in Figures 8A, B, high-risk patients exhibited a higher frequency of gene mutations with TP53 mutation frequency being notably elevated compared to low-risk patients. Meanwhile, higher TMB scores were observed in high-risk patients, increasing alongside risk score elevation (Figures 8C, D). Moreover, patients with higher TMB scores experienced poorer OS (*p*-value<0.031), and those with both higher TMB scores and risk scores exhibited the worst OS (*p*-value<0.001) (Figures 8F, G). Moreover, a correlation analysis has been conducted for assessing the relationship between immune checkpoint gene expression and the prognostic signature. The expression of 29 ICGs were observed to be significantly correlated with the risk score (Supplementary Table S20), with 21 displaying positive correlations and eight exhibiting negative correlations (Figure 8E). In total, nine ICGs with expression levels positively correlated with the risk score were selected for visualization and survival analysis based on their correlation coefficients (Figure 8H, Supplementary Figure S10). The findings indicated patients with higher risk scores and higher elevated levels of eight of the aforementioned genes experienced worse OS. However, patients with higher risk scores and lower TNFRSF9 expression levels exhibited poorer OS.

### Confirming the predictive value of the prognostic signature in the immunotherapy response for HCC

**FIGURE 9:**
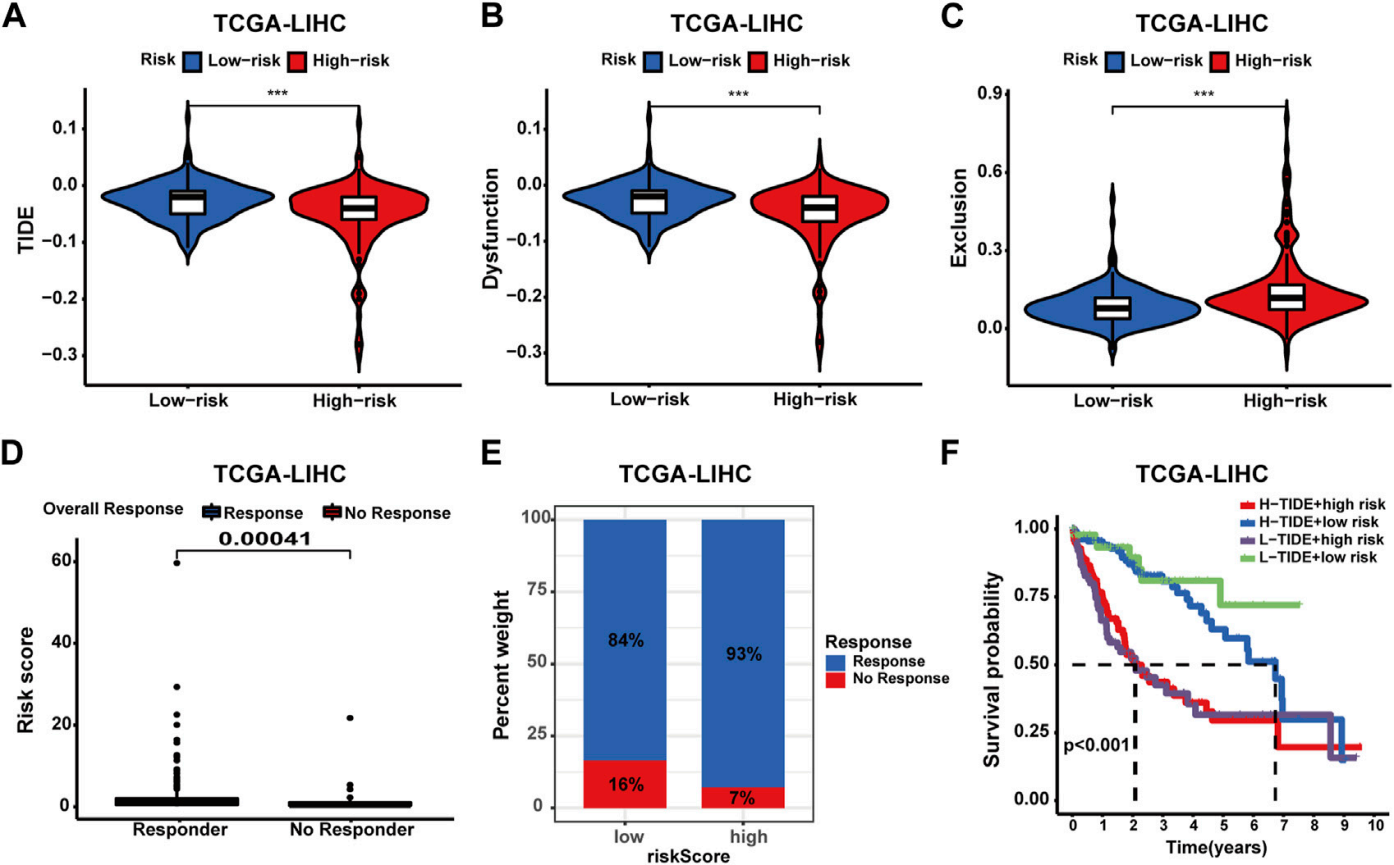
Assessing the predictive value of the prognostic signature in immunotherapy response for HCC patients of the TCGA cohort. **(A)** TIDE score between patients with different risk profiles. **(B)** Dysfunction score between patients with different risk profiles. **(C)** Exclusion score between patients with different risk profiles. **(D)** Correlation analysis of the risk score and immunotherapy response. **(E)** Distribution of immunotherapy response in patients with different risk profiles. **(F)** Survival analysis of patients in four groups categorized by the risk score and TIDE score.

**FIGURE 10:**
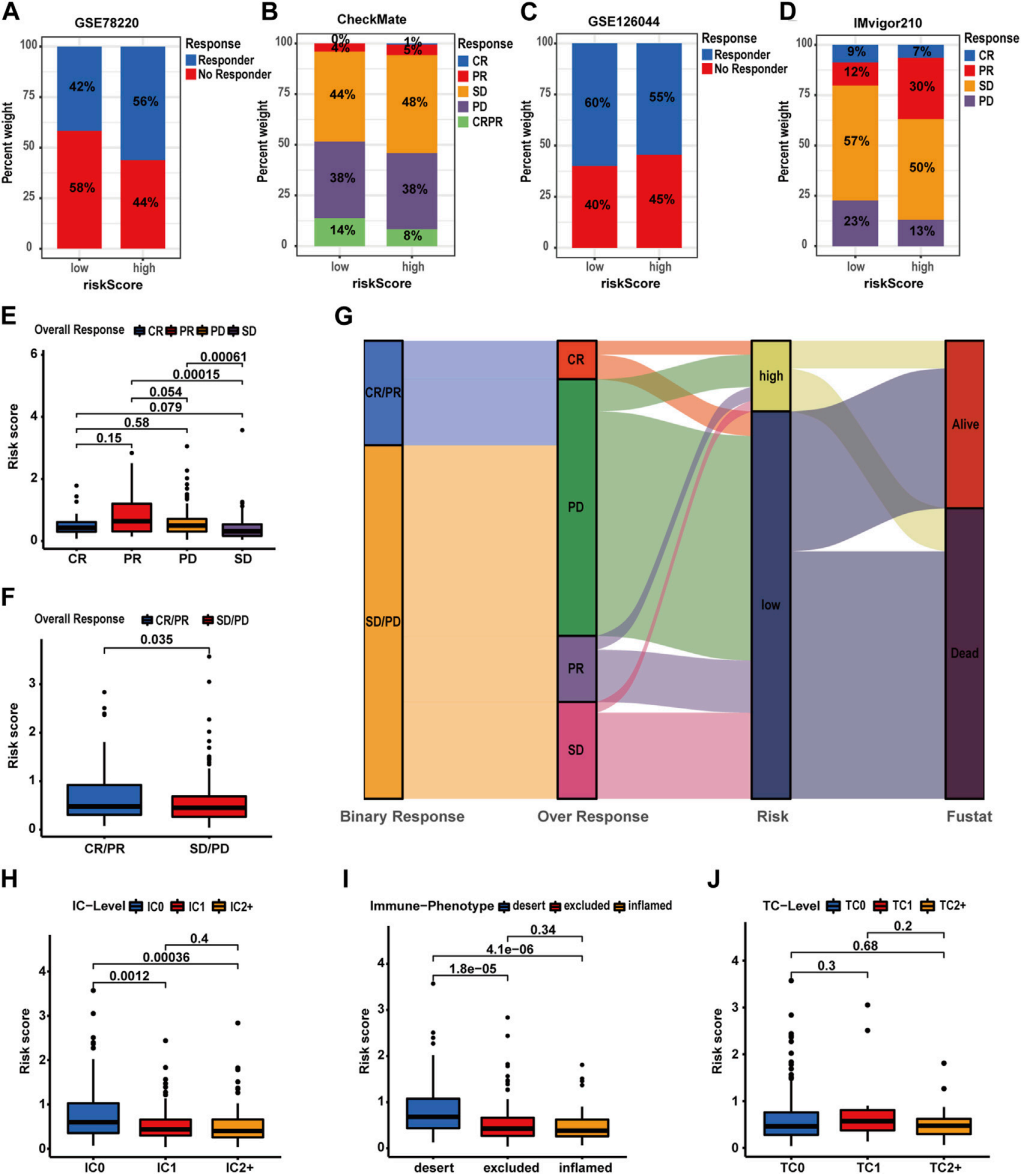
Validating the predictive value of the prognostic signature in immunotherapy response for HCC patients of more cohorts. **(A**–**D)** Distribution of immunotherapy response in patients with different risk profiles. **(E**–**F)** Correlation analyses of the risk score and immunotherapy response in the IMvigor210 cohort. **(G)** Correlations of the prognostic signature with binary response, over-response, and survival status in the IMvigor210 cohort. **(H**–**J)** Correlation analysis of the risk score, IC level, TC level, and immune phenotypes in the IMvigor210 cohort.

To comprehensively evaluate the predictive capacity of the prognostic signature for immunotherapy responsiveness, the TIDE algorithm was utilized. High-risk patients exhibited a lower TIDE score (*p* < 0.001), signifying a heightened probability of immunotherapy responsiveness (Figure 9A). Concurrently, these patients demonstrated increased exclusion scores and diminished dysfunction scores (Figures 9B, C). Moreover, superior overall response rates were observed among high-risk patients (Figures 9D, E). Furthermore, patients with higher risk scores and elevated TIDE scores experienced the most unfavorable overall survival outcomes (Figure 9F). Next, four independent cohorts were employed for validation purposes. In the GSE78220 and IMvigor210 cohorts, immunotherapy response rates among high-risk patients were elevated, consistent with prior findings; however, no discernable differences were observed in the remaining two cohorts (Figures 10A–F). A Sankey diagram was employed to delineate the distribution of immunotherapy responses in patients with different risk levels and survival statuses within the IMvigor210 cohort (Figure 10G). In the same cohort (IMvigor210), we explored the correlation among the risk score, immune phenotypes, tumor cell levels (TC-levels), and immune cell levels (IC-levels) (Figures 10H–J). Herein, a preliminary correlation between immune infiltration levels and risk scores is delineated.

### Evaluation of the predictive value of the prognostic signature in drug sensitivity of HCC patients

**FIGURE 11:**
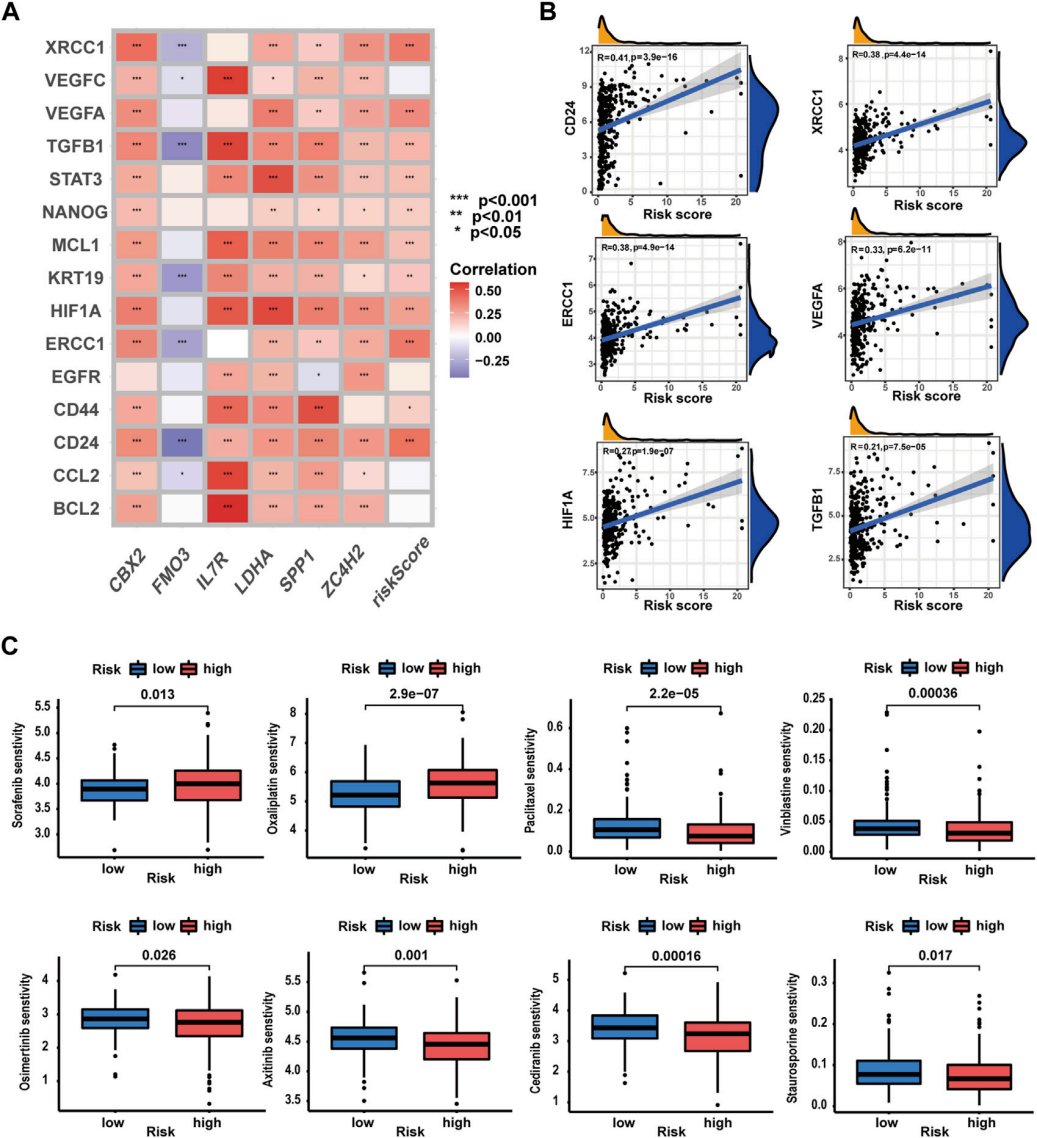
Evaluation of the predictive value of the prognostic signature in drug sensitivity of HCC patients. **(A)** Correlations analysis of the prognostic signature and CRRG expression patterns. **(B)** Correlation analysis of CRRG expression levels with the risk score. **(C)** Drug sensitivity between patients with different risk profiles (*p-value <0.05, **p-value <0.01, and ***p-value <0.001).

In this part, we evaluated the prognostic signature’s performance in predicting drug susceptibility. Expression levels of 11 CRRGs demonstrated a positive correlation with risk scores (Supplementary Table S21) (Figure 11A). A visualization featuring nine CRRGs was generated based on correlation coefficients (Figure 11B). Subsequently, we ascertained the predictive value of the prognostic signature for drug sensitivity via the R package “oncoPredict.” Lower sensitivity scores signified enhanced drug responsiveness in HCC patients. Figure 11C reveals that high-risk patients exhibit heightened sensitivity to agents such as paclitaxel, cediranib, and osimertinib, while low-risk patients may demonstrate favorable responses to sorafenib and oxaliplatin. These findings suggest that the prognostic signature holds potential for predicting chemosensitivity in patients and informing HCC chemotherapy strategies.

### Validation of prognostic signature genes

**FIGURE 12:**
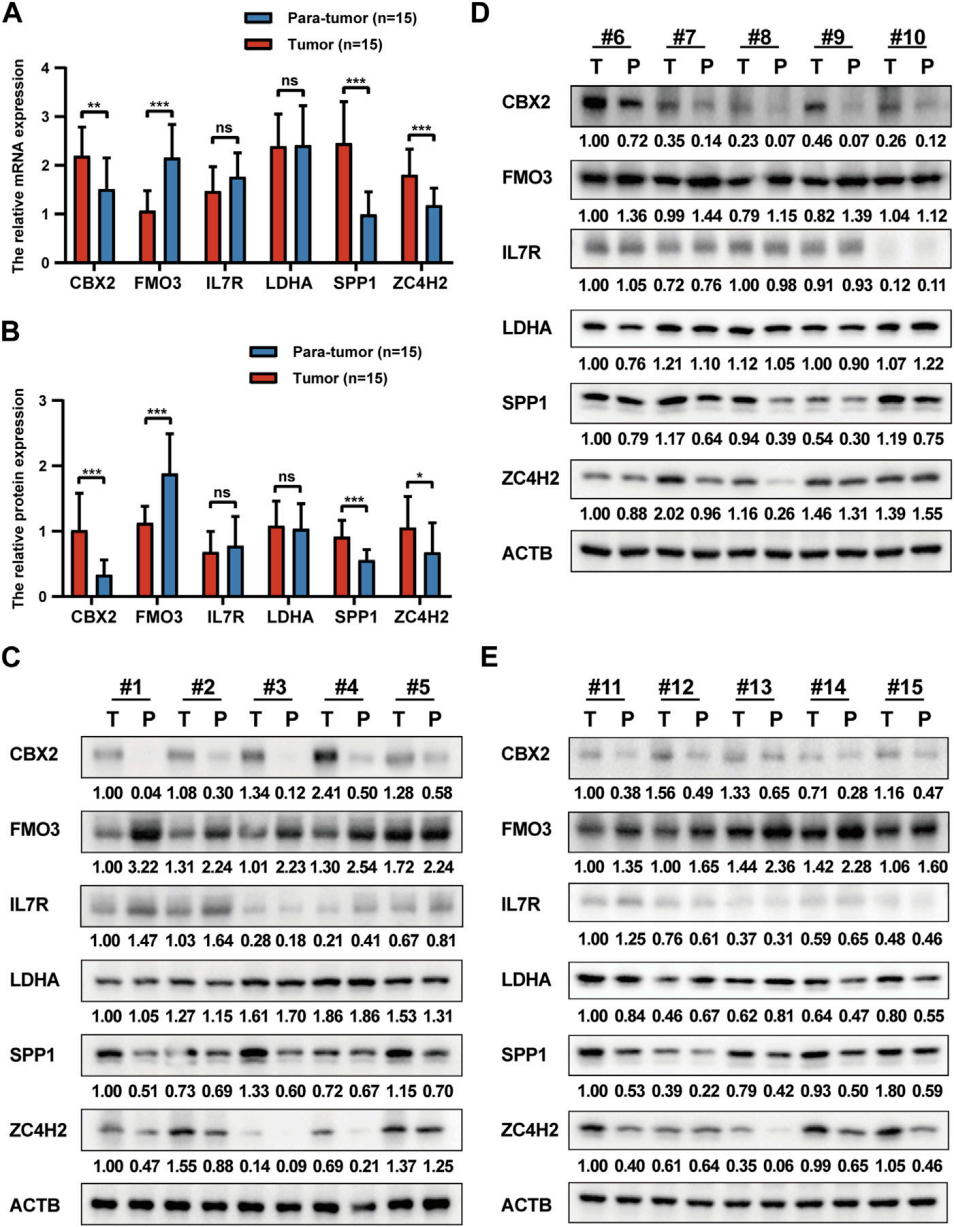
Validation of prognostic signature genes. **(A)** The relative expression levels of six prognostic signature genes in HCC tumor tissues and para-tumor tissues at the mRNA level. **(B)** The relative expression levels of six prognostic signature genes in HCC tumor tissues and para-tumor tissues at the protein level. **(C–E)** The WB results of six prognostic signature genes.

Here, we verified the expression levels of prognostic signature genes in HCC clinical tissue samples by qRT-PCR and Western blot analyses. The qRT-PCR results revealed a significant upregulation of CBX2, SPP1, and ZC4H2, alongside a notable downregulation of FMO3 in tumor tissues. Conversely, no significant differences were observed in IL7R and LDHA expression (Figure 12A). Consistent findings were obtained from WB analysis. Significant disparities in protein expression levels of CBX2, SPP1, ZC4H2, and FMO3 were detected between tumor tissues and matched para-tumor tissues. Simultaneously, heterogeneous IL7R expression was identified, with a marked upregulation at the protein expression level of IL-7R in six paired clinical samples and no significant differences in the remaining samples (Figures 12B–E).

## Discussion

Nowadays, HCC poses a significant challenge to global health due to the rising incidence and mortality rates, compounded by the scarcity of effective treatment options (Villanueva, 2019; Vogel et al., 2022). Recently, the integration of bioinformatics and omics technologies has established the groundwork for precision medicine in HCC management. High-throughput molecular profiling engenders optimistic expectations for the identification of novel therapeutic targets and biomarkers, thereby catalyzing advancements in targeted and immunotherapeutic approaches for HCC treatment (Forner et al., 2018). However, it remains an intractable issue of resistance to the aforementioned therapies in HCC, potentially correlated with the inherent apoptotic resistance exhibited by cancer cells (Mohammad et al., 2015; Llovet et al., 2018). Programmed cell death serves as the principal mechanism for eliminating aberrant cells within the human body, playing a crucial role in biological processes. Simultaneously, an extensive corpus of the scientific literature corroborates the pivotal role of PCD in the initiation and advancement of oncogenic malignancies (Peng F. et al., 2022). Cellular apoptosis represents one of the most archetypal programmed cell death pathways. Since its discovery, apoptosis has been harnessed as a primary method for cancer therapy by promoting the apoptotic demise of malignant cells (Singh et al., 2019). However, considering apoptotic resistance, targeting alternative non-apoptotic programmed cell death pathways may prove to be a more efficacious approach (Mohammad et al., 2015; Carneiro and El-Deiry, 2020). Ferroptosis, a novel non-apoptotic programmed cell death, not only directly inhibits tumor progression but also exerts antitumor effects by modulating the tumor microenvironment. Studies have demonstrated that in transgenic mouse models of lung cancer and HCC, inducing ferroptosis in tumor-associated macrophages effectively suppresses tumor formation and metastasis, while enhancing the efficacy of anti-PD-L1 therapy (Tang et al., 2023a; Tang et al., 2023b). In HCC, pyroptosis primarily exerts antitumor effects (Zou et al., 2023). Sorafenib was reported to inhibit HCC progression by inducing macrophage pyroptosis and enhancing the tumoricidal activity of NK cells (Hage et al., 2019). Cuproptosis and necroptosis, as previously mentioned, are closely related to the development of HCC. Notably, before cuproptosis was identified as an independent cell death form highly associated with mitochondrial respiration and the lipoic acid (LA) pathway, the link between copper ions and tumor progression and programmed cell death had already attracted the attention of researchers. Researchers previously attributed this cell death to copper’s effect on mitochondria, resulting in ROS production, which is highly similar to ferroptosis, until Tsvetkov et al. (2022) confirmed the unique mechanism of copper-induced cell death. Intriguingly, numerous investigations have revealed that diverse programmed cell death pathways form intricate networks of cellular demise, characterized by their adaptability and interplay with one another. In addition, it is possible to identify central signaling hubs for diseases by investigating the joint role of multiple PCD pathways (Bedoui et al., 2020). Although no reports currently exist regarding the potential synergistic effects of cuproptosis and necroptosis in tumor initiation and progression, we have noted evidence suggesting crosstalk between these two cell death modalities at the level of regulatory factors and signaling pathways. XIAP, a potent apoptotic suppressor, is involved in the regulation of both necroptosis and cellular copper homeostasis, thereby affecting cuproptosis (Burstein et al., 2004; Wicki et al., 2016). The regulation of cuproptosis is associated with numerous oncogenic signaling pathways because copper is directly binding and activating key molecules within these pathways. These pathways primarily are PI3K-AKT, MAPK, autophagy, HIF-1α, NF-κB, and notch signaling pathways (Xie et al., 2023). Among these, the HIF-1α and NF-κB signaling pathways play crucial roles in the regulation of necroptosis (Dondelinger et al., 2015; Karshovska et al., 2020). These observations suggest a connection between cuproptosis and necroptosis at the molecular basis and signaling regulation levels, thus providing insights for our study. Subsequently, we ascertained 29 CRNGs to elucidate the concomitant roles of cuproptosis and necroptosis in HCC. These genes were discerned via correlation analysis premised on the expression of CRGs and NRGs in HCC patients, adhering to the criteria of |R|>0.6 and *p*-value <0.001. An exhaustive investigation of gene mutation characteristics and CRNG expression patterns was conducted, leading to the development of a CRNG subtype-related prognostic signature comprising six pivotal genes. Subsequently, clinical nomograms integrating the prognostic signature and clinicopathological features were devised for HCC patients. Collectively, the CRNG subtype-related prognostic signature exhibited satisfactory efficacy in prognostic prediction and clinical application. In this study, we initially delineated the mutational and expression landscapes of CRNGs. The results revealed mutations in CRNGs among 61 patients with CDKN2A and NFE2L2 displaying the highest mutational frequency. It is noteworthy that CDKN2A and NFE2L2 are concurrently involved in both cuproptosis and necroptosis. Moreover, 17 CRNGs were upregulated in tumor tissues, while only AIM2, AXL, and TLRL4 were downregulated. Concurrently, we found that elevated CRNG expression predominantly correlated with unfavorable HCC prognosis. Based on CRNG expression patterns, patients were stratified into two distinct subtypes with patients in subtype B experiencing inferior prognoses and elevated CRNG expression. To explore potential treatment response disparities between the two subtypes, we compared chemoresistance-related gene and immune checkpoint gene expression levels. In total, 18 differentially expressed CRRGs were identified, implicating diverse mechanisms of chemoresistance in HCC. For example, aberrant EGFR activation mediates sorafenib resistance in HCC, which may be potentiated by an EGFR-KLF4 positive feedback loop (Blivet-Van Eggelpoël et al., 2012; Pang et al., 2019), while polymorphisms in VEGFA and VEGFC similarly confer sorafenib resistance (Scartozzi et al., 2014). Also, significant disparities in ICG expression were observed between the two subtypes. Subsequently, we evaluated immune cell infiltration disparities among patients, identifying significant differences in activated CD4^+^ T-cell, activated dendritic cell, and Treg infiltration levels between the subtypes, potentially contributing to divergent clinical outcomes here. CD4^+^ T cells are crucial for suppressing HCC progression (Ma et al., 2016). Dendritic cells function as vital antitumor immune cells, whose depletion impairs CD8^+^ T-cell recruitment and immune checkpoint therapy responsiveness (Salmon et al., 2016). Tregs are implicated in establishing an immunosuppressive tumor microenvironment and engendering chemoresistance in HCC (Liu et al., 2022). In summary, our findings here underscore a potential association between CRNG expression, clinical features, and immune cell infiltration levels, indicating a cooperative contribution of cuproptosis and necroptosis in shaping the immune TME and regulating HCC progression.

Next, a comprehensive functional enrichment examination was performed. Through GSVA and GSEA analysis, we identified conspicuous disparities in processes linked to protein degradation and mitochondrial function among patients from two distinct subtypes, such as ubiquitin-mediated protein degradation, proteasome and lysosome function, and oxidative phosphorylation function, which corrected with cuproptosis (Tsvetkov et al., 2022). In addition, variations in processes pertinent to necroptosis, including inflammatory responses, TNFA, and TLR signaling pathways, were observed (Seo et al., 2021). Furthermore, we discerned numerous cancer-related biological functions and signaling pathways enriched in patients of subtype B, such as p53, Wnt/β-catenin signaling pathways, epithelial–mesenchymal transition (EMT), angiogenesis, and cell cycle regulation, potentially resulting in a dismal prognosis for patients of subtype B. Analogous outcomes were attained via GO/KEGG analyses. The differences were observed in cancer-associated signaling pathways, cellular proliferation and division (e.g., chromosomal segregation, DNA replication, and mitosis), protein degradation, inflammatory response, energy metabolism, etc. These results revealed potential mechanisms underlying HCC progression, mediated conjointly by cuproptosis and necroptosis, and demonstrate the utility of CRNGs for characterizing their synergistic role. In a word, this section provides preliminary evidence for the synergistic involvement of cuproptosis and necroptosis in the pathogenesis and progression of HCC. The cuproptosis-related necroptosis genes may possess the capacity to concurrently regulate cuproptosis and necroptosis in HCC, thereby further orchestrating the progression of HCC. This finding offers critical insights into the cooperative roles of cuproptosis and necroptosis in hepatocellular carcinoma, thereby facilitating a deeper understanding of these two non-apoptosis pathways in HCC development. In order to further investigate the roles of CRNG subtypes, we performed a transcriptomic analysis and identified 3,855 OS-DEGs by univariate Cox regression. We then discovered three OS-DEG expression patterns associated with clinical characteristics. Next, we constructed a CRNG subtype-related prognostic signature composed of CBX2, FMO3, IL7R, LDHA, SPP1, and ZC4H2. CBX2, a constituent of PcG complexes, has been implicated in the facilitation of HCC cell proliferation (Mao et al., 2019). FMO3, an inhibitory factor in HCC, has been observed to enhance the survival and growth of HCC cells upon downregulation (Hlady et al., 2019). For IL7R, a recent investigation revealed that the hepatitis B virus could augment HCC cell proliferation and migration by elevating IL7R expression levels (Kong et al., 2016). In HCC, LDHA silencing has been demonstrated to impede tumor development and promote CD4^+^ T-cell infiltration (Serra et al., 2022). SPP1, which contributes to tumor cell evolution and TME reprogramming, has been reported to be a valuable therapeutic target for HCC (Ma et al., 2021). The role of ZC4H2 in HCC is still unclear, and our investigation may illuminate the potential significance of ZC4H2 in this context. Through qRT-PCR and WB analyses, we furnished additional substantiation, underscoring the plausible functions of these signature genes in HCC, with particular emphasis on ZC4H2. Here, our findings demonstrated an upregulation of ZC4H2 expression in HCC tumor tissues, insinuating its potential involvement in accelerating HCC progression. Furthermore, through functional enrichment analysis, we found a significant correlation between four signature genes (CBX2, LDHA, SPP1, and ZC4H2) and malignant tumor biology, exhibiting upregulation in high-risk patients. Conversely, FMO3 and IL7R, downregulated in high-risk patients, were implicated in multiple metabolic pathways, inflammatory responses, and immune processes. Enhanced metabolism has been associated with improved prognoses in HCC patients, suggesting a potential mechanism by which FMO3 may hinder HCC progression (Gao et al., 2019). Although IL-7R has been identified as a risk factor for hepatitis B virus (HBV)-related HCC in previous studies, some comprehensive studies have revealed its potential protective role in HCC cases beyond solely HBV-related instances (Kong et al., 2016; Yin et al., 2020; Zhuang et al., 2021). Our investigation supports IL-7R as a protective factor in HCC, despite observing no significant differences in expression levels between tumor and para-tumor tissues. The confusing role of IL7R may be attributable to heterogeneous expression patterns among distinct HCC patient populations (HBV-related HCC patients and non-viral-related HCC patients), and more clinical samples are needed for the specific role of IL-7R in HCC. Based on the CRNG subtype-related prognostic signature, we found that HCC patients with elevated risk scores suffered poorer OS and had higher CRNGs expression levels, consistent with those who were in CRNG subtype B or gene subtype B. Furthermore, a correlation analysis among risk scores, CRNG subtypes, gene subtypes, and survival status was conducted, verifying the consistency and stability of this signature in prognostic prediction. With advanced evaluation and validation, we confirmed the precision and applicability of the CRNG subtype-related prognostic signature. Survival curves and distribution plots demonstrated the ability of this signature in predicting and discriminating the prognosis for HCC patients. ROC curves exhibited high accuracy of the signature in predicting outcomes for HCC patients. Univariate and multiple regression analysis and C-index analysis established the CRNG subtype-related prognostic signature as a strong independent prognostic determinant for HCC patients. Subsequently, we developed clinical nomograms incorporating the CRNG subtype-related prognostic signature and clinicopathological features of HCC patients, facilitating the signature’s practical implementation in clinical settings. The nomograms’ accuracy was appraised using calibration and ROC curve analyses. Subsequently, we conducted a comprehensive analysis encompassing multiple dimensions to scrutinize potential factors associated with divergent prognoses among patients with two risk profiles. The HCC immune microenvironment is a complex mixture consisting of stromal cells, tumor cells, and immune cells. The infiltration of particular immune cells imparts immunosuppressive attributes in HCC, with the archetypal instances being Tregs and TAMs, which confer an unfavorable prognosis (Sangro et al., 2021). For the results, we found an upregulation of inflammation-associated immune functionality and elevated stromal estimate scores in low-risk patients. We also observed a positive correlation between high- risk scores and immune cell infiltration, such as monocytes, M0, M2-TAMs, neutrophils, Tregs, and naive B cells, while M1-TAMs, NK cells, and T lymphocytes, excluding Tregs, were associated with lower risk scores. It has been reported that neutrophil not only potentiates HCC progression by mediating TAMs and FOXP3^+^ Tregs infiltration but also induces resistance to sorafenib (Zhou et al., 2016). Naive B cells have been demonstrated to impede HCC development, with diminished infiltration levels in HCC and higher infiltration levels signifying improved prognoses and T-cell activation (Zhang et al., 2019). The abundance and functionality of NK cells are positively correlated with prognosis but their suppression frequently occurs in HCC (Cai et al., 2008). These findings indicated that the formation of an immunosuppressive microenvironment could precipitate unfavorable prognoses in high-risk patients. Concurrently, we identified a positive correlation between risk scores and stemness scores, which could engender poor prognoses in HCC patients (Lee et al., 2022). Moreover, high-risk patients exhibited enrichment in cancer-associated signaling pathways, cell cycle regulation, protein degradation, genomic stability, nucleotide metabolism, and energy metabolism, insinuating the potential molecular mechanisms correlating with poor prognosis. Finally, we probed the predictive capacity of the CRNG subtype-related prognostic signature in treatment response. TMB has been linked to the prognosis of various cancer patients and immune checkpoint therapy outcomes (Valero et al., 2021). In our study, we observed an elevated gene mutation frequency in high-risk patients, with TP53 mutation frequency exhibiting the most pronounced disparity between the two patient groups. Mutation in the *TP53* gene represents a prevalent event in HCC, thus culminating in advanced clinicopathological attributes and poor prognosis (Calderaro et al., 2017). Meanwhile, our results exhibited a correlation between elevated TMB scores and poor prognosis, indicating immune checkpoint therapy could potentially ameliorate the unfavorable prognosis of high-risk patients. In addition, 29 ICGs were identified as correlating with the CRNG subtype-related prognostic signature, suggesting their potential as predictive markers for immune checkpoint therapy also. These results above showed the preliminary evidence in the predictive value of the signature for immune checkpoint therapy, and a further validation has been performed by TIDE algorithms. In TCGA-LIHC and another four cohorts, we observed a higher treatment response rate in high-risk patients, underscoring the signature’s predictive capacity and its applicability in guiding immune checkpoint therapy for HCC. Similarly, the CRNG subtype-related prognostic signature could be applied to guide chemotherapy for HCC. With the signature, we could forecast drug sensitivity in patients of two groups. It was shown that paclitaxel, cediranib, and osimertinib were sensitive in high-risk patients, whereas sorafenib and oxaliplatin were sensitive in other patients. Moreover, CRRGs exhibited significant associations with the CRNG subtype-related prognostic signature, suggesting the signature could be a biomarker for chemoresistance in HCC patients. Overall, our study unveiled the role of synergizing cuproptosis and necroptosis in HCC for the first time. The CRNG subtype-related prognostic signature possesses potential utility for both prognosis prediction and therapeutic guidance in HCC patients. However, several caveats warrant attention in this study. First, we constructed the CRNG subtype-related prognostic signature based on public databases. We need more data from prospective studies to confirm these outcomes. Also, more clinical tissue samples and verification experiments are needed to explore the functions of signature genes in HCC. Second, several samples were excluded because of incomplete data, which could affect the accuracy of subsequent analysis. Third, we introduced multiple cohorts to further validate the predictive value in the CRNG-related prognostic signature of immune checkpoint therapy response, but not all the cohorts are based on HCC; more evidence is needed.

## Conclusion

This investigation established a CRNG subtype-related prognostic signature, thus exhibiting significant associations with overall survival, clinicopathological traits, tumoral immune microenvironment, stemness attributes, tumor mutational burden, and treatment susceptibility. This signature possesses potential utility for prognostic forecasting and therapeutic direction in HCC.

## Data Availability

The original contributions presented in the study are included in the article/Supplementary Material; further inquiries can be directed to the corresponding authors.
