# Supplementary figures and images for: Identifying a novel cuproptosis-related necroptosis gene subtype-related signature for predicting the prognosis, tumor microenvironment, and immunotherapy of hepatocellular carcinoma
Source: Front Mol Biosci. 2023 May 23;10:1165243. doi: 10.3389/fmolb.2023.1165243 (PMC10242026; doi:10.3389/fmolb.2023.1165243)

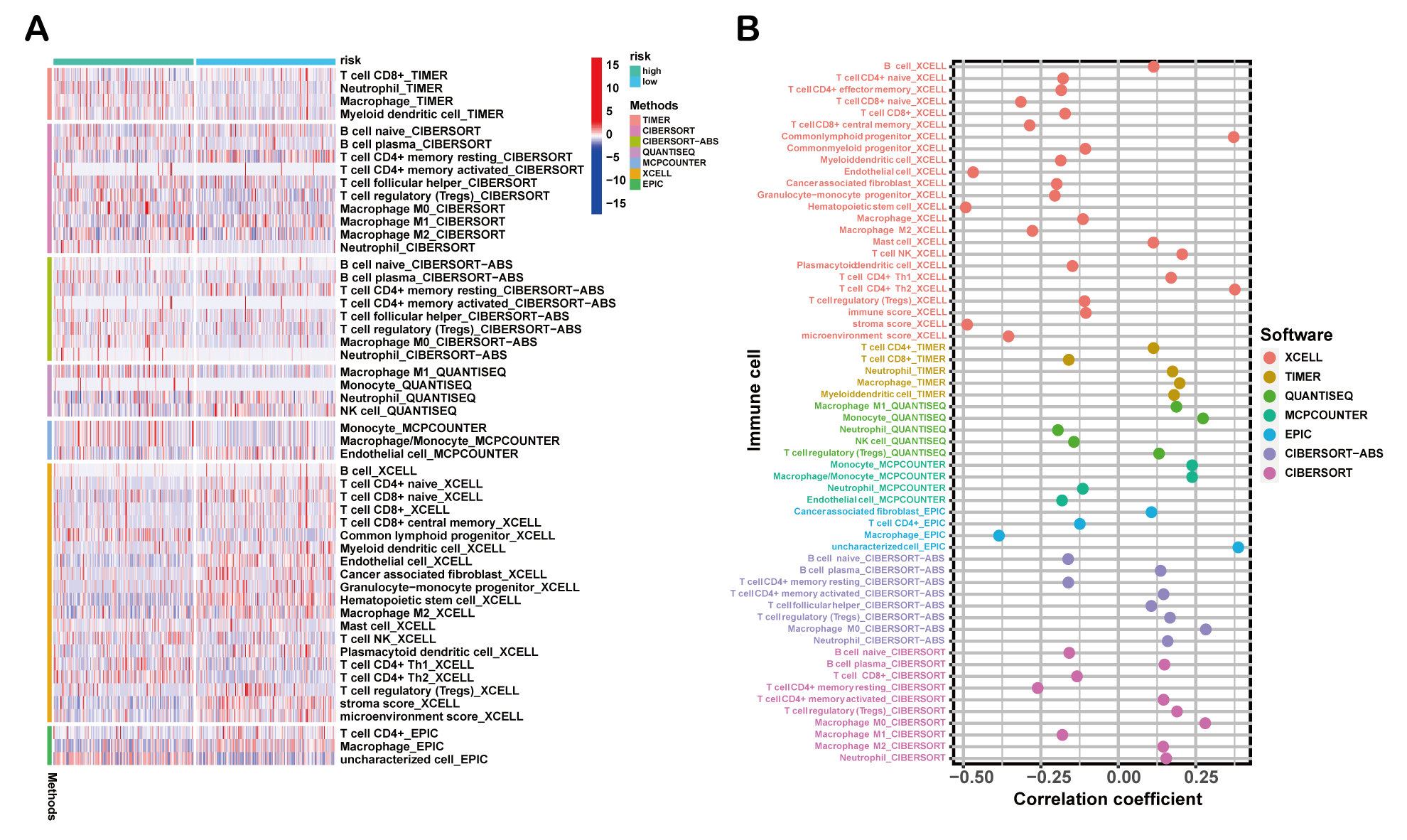

Supplement: Supplementary file 2 [file Image6.TIF]

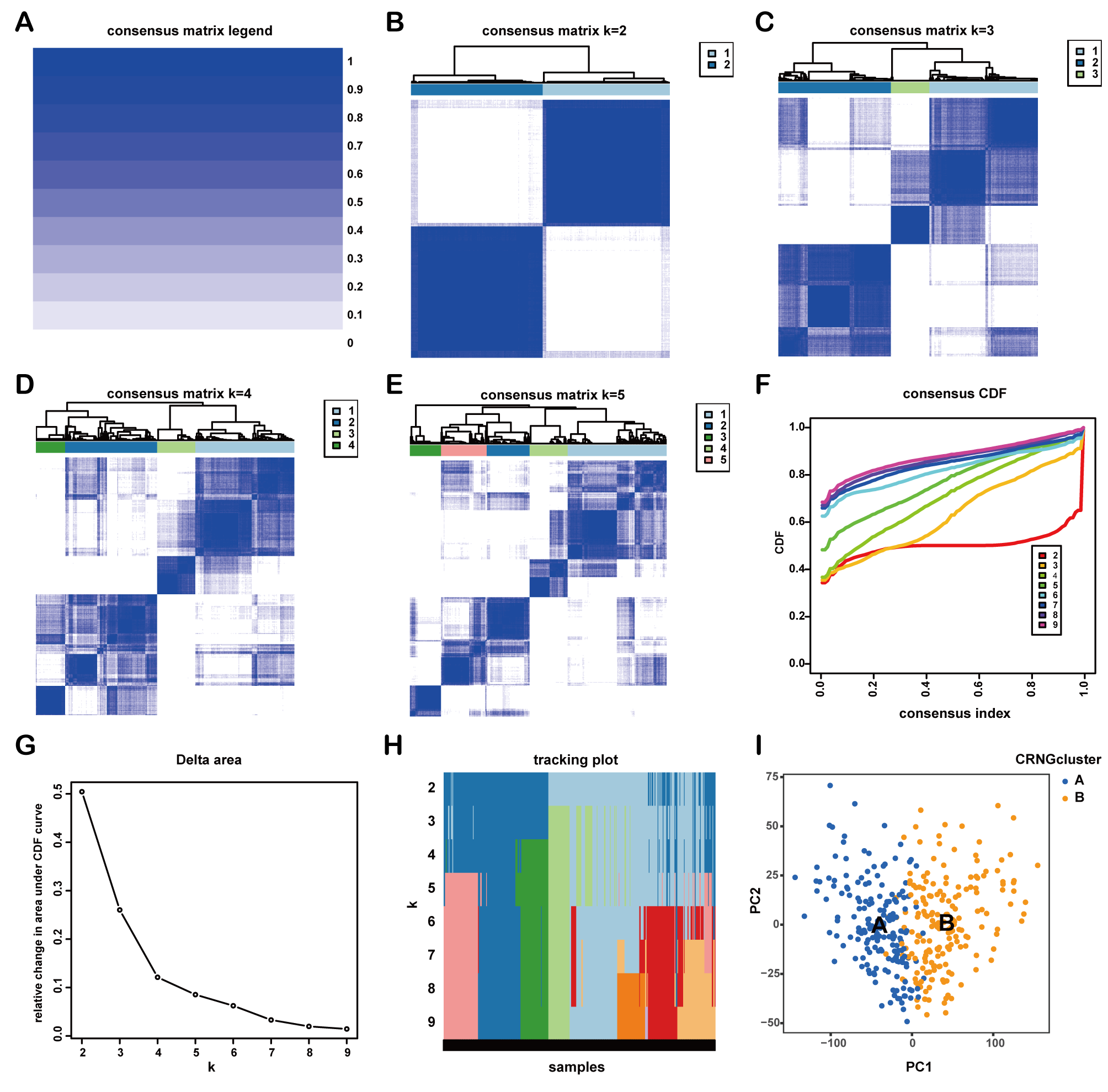

Supplement: Supplementary file 3 [file Image3.TIF]

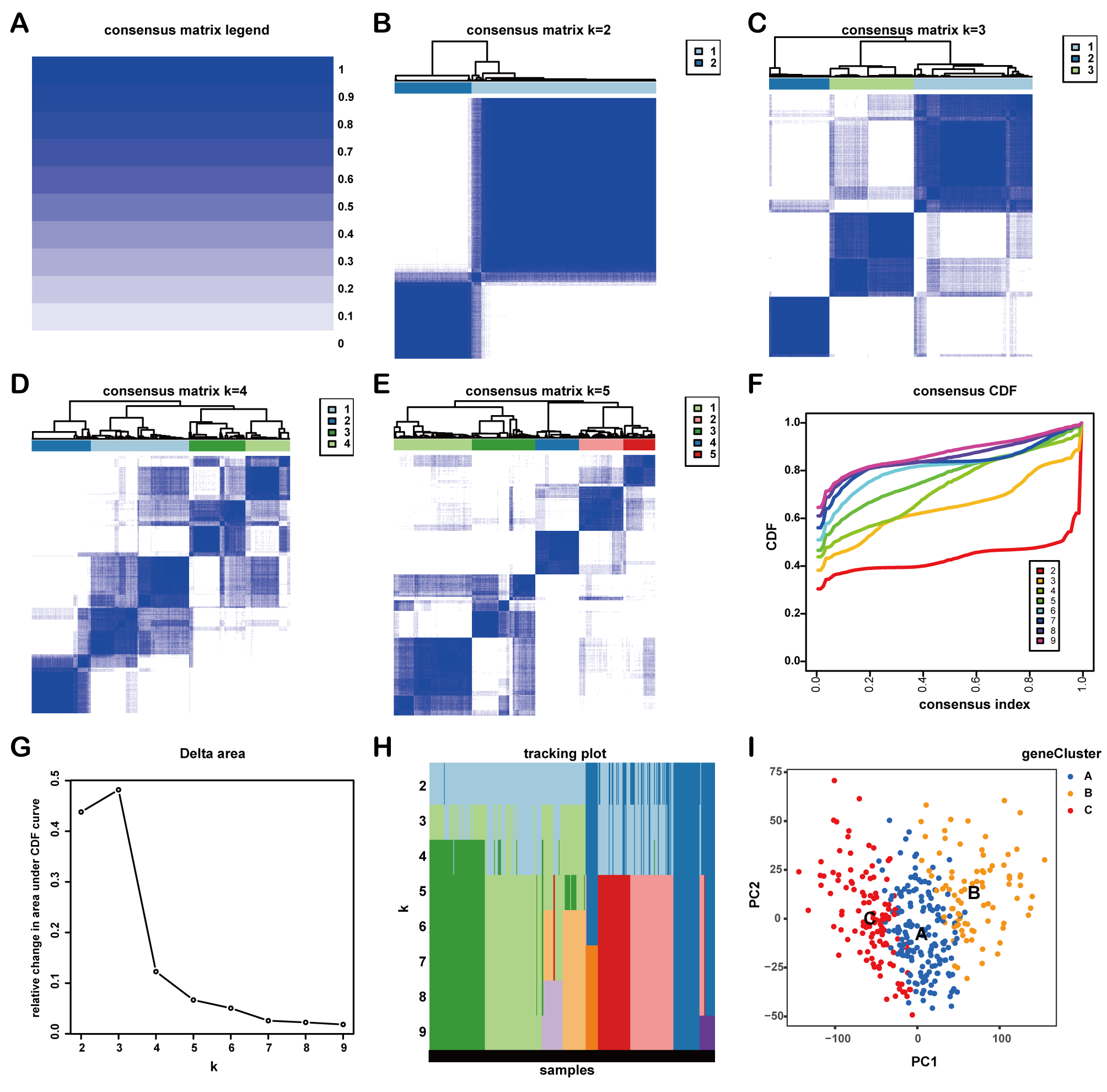

Supplement: Supplementary file 4 [file Image4.TIF]

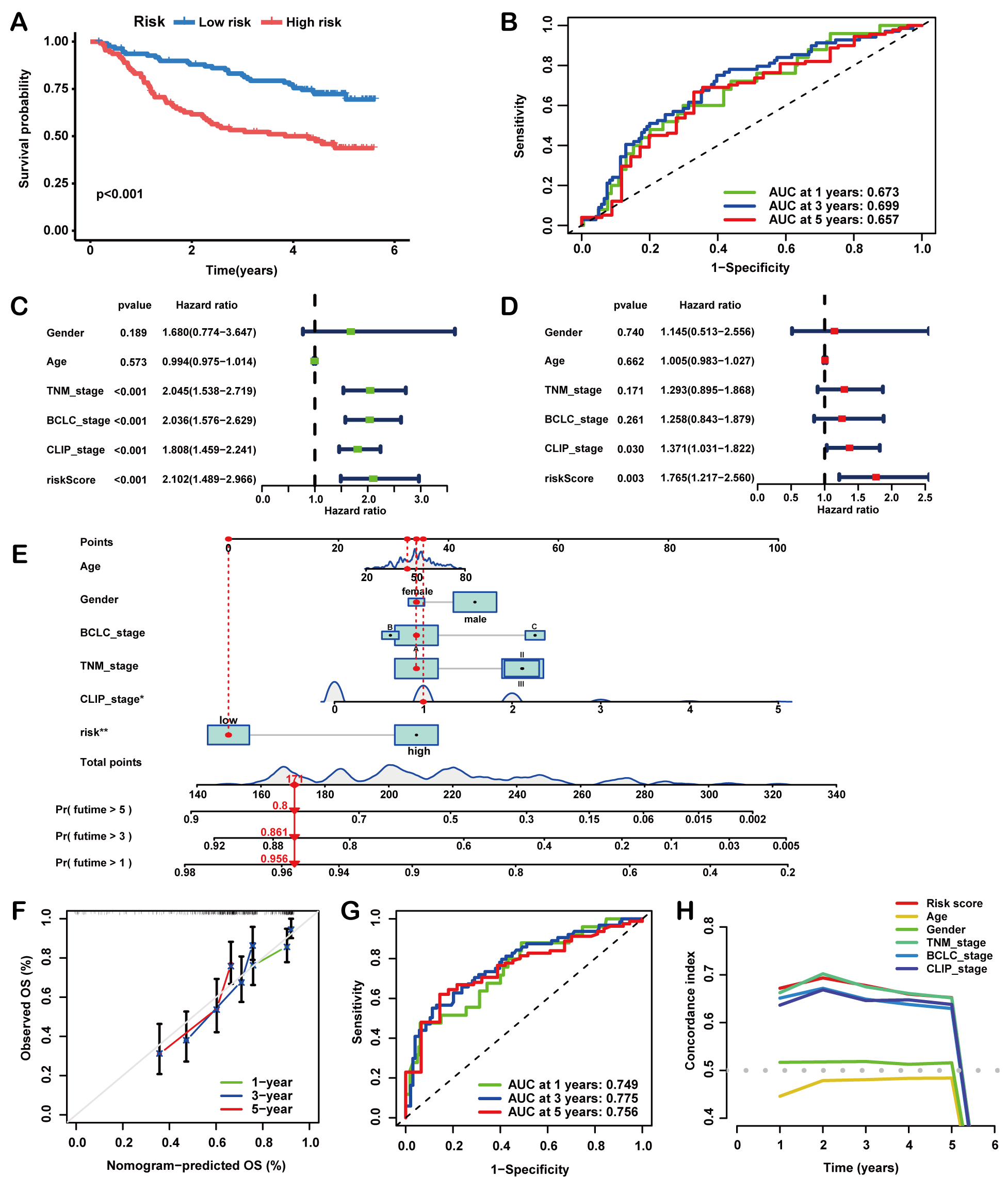

Supplement: Supplementary file 5 [file Image9.TIF]

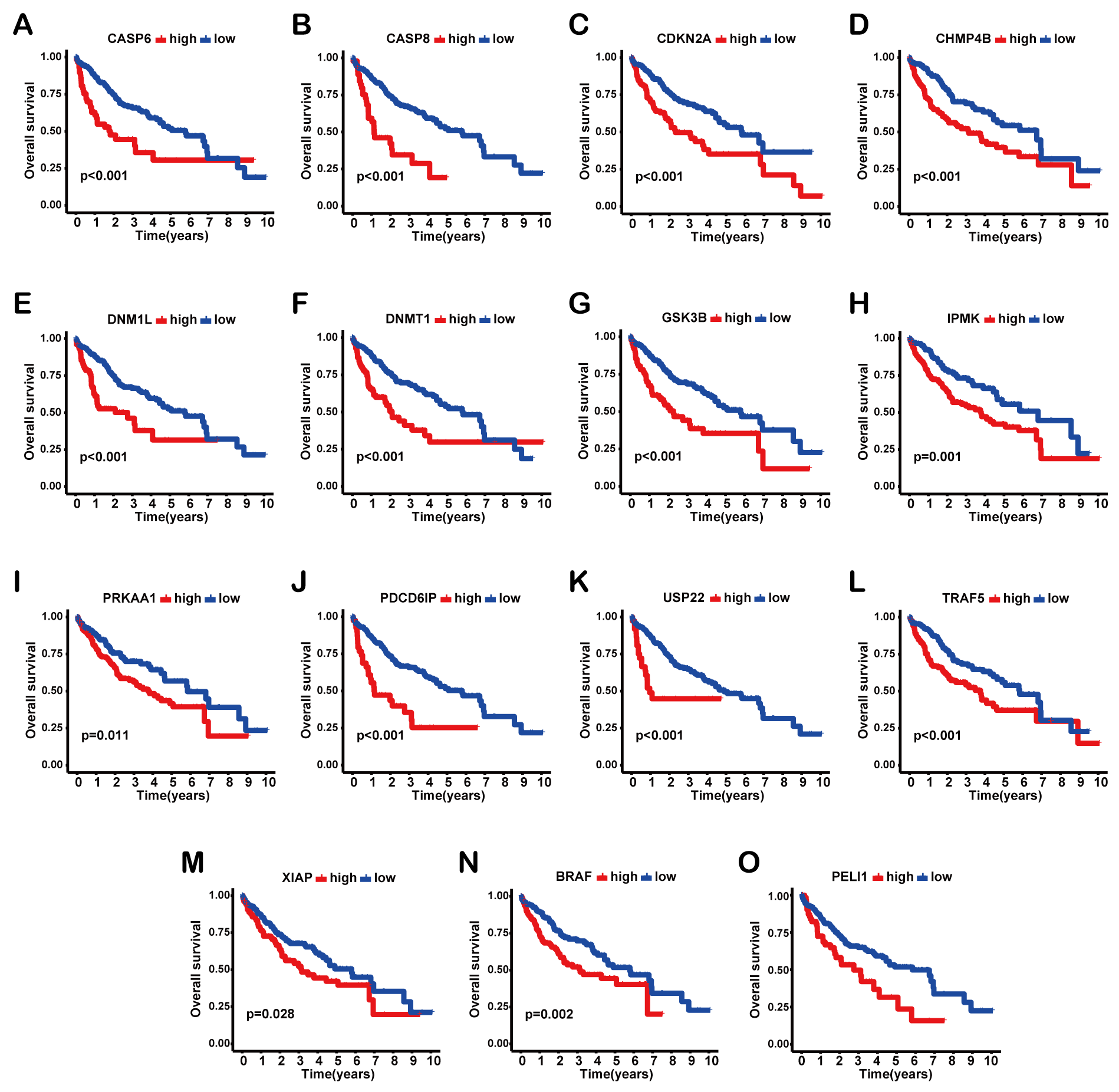

Supplement: Supplementary file 6 [file Image2.TIF]

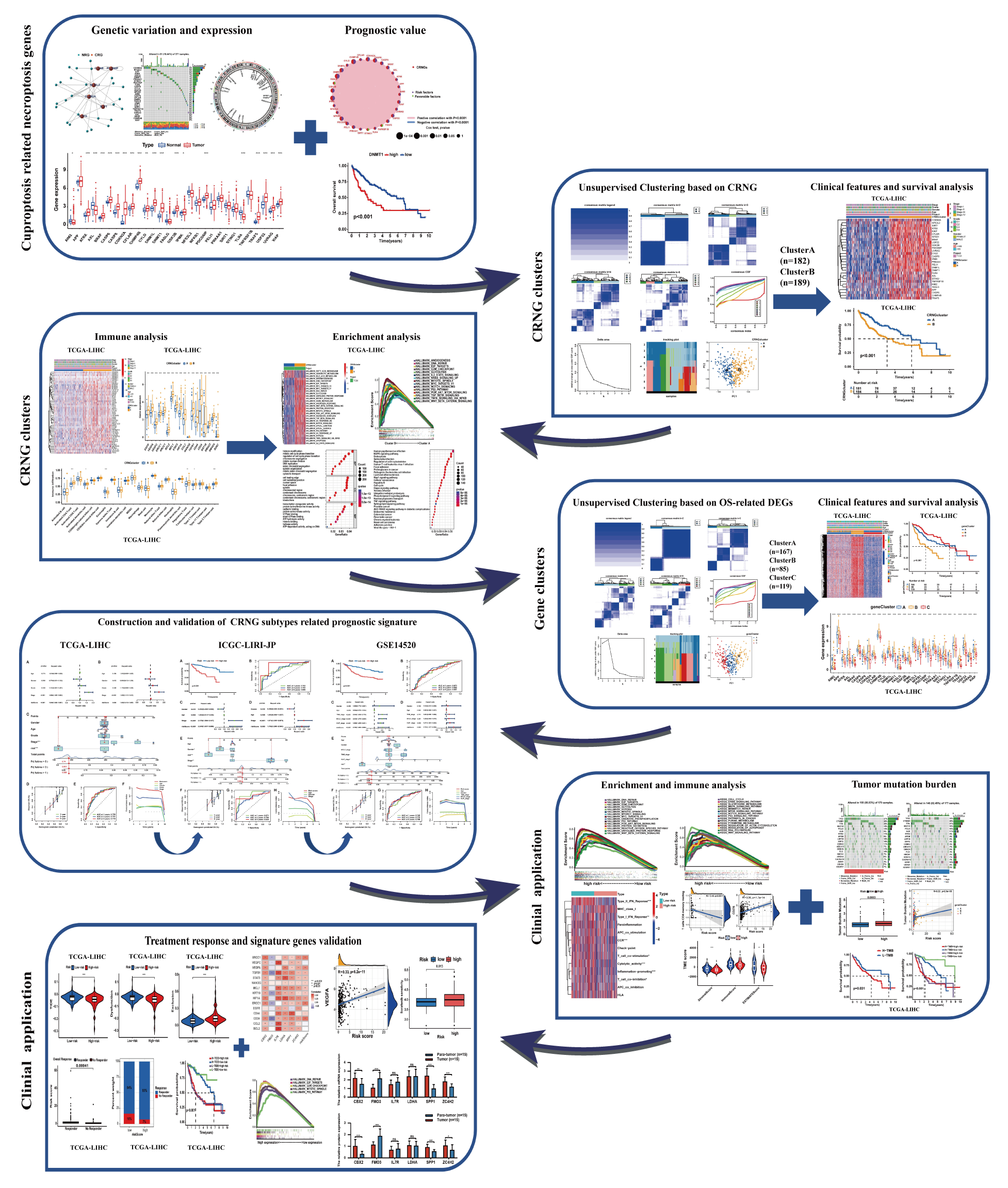

Supplement: Supplementary file 7 [file Image1.TIF]

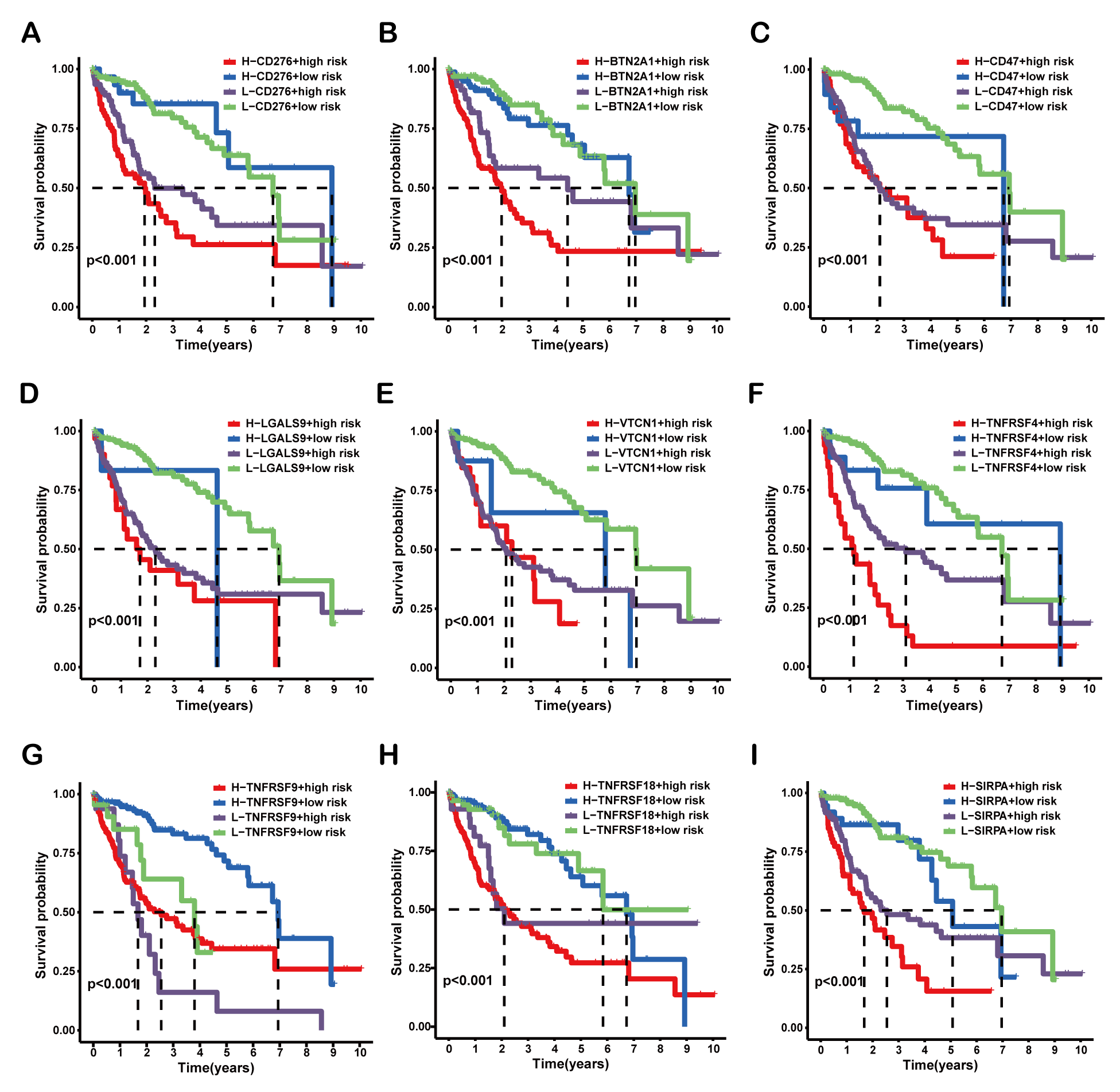

Supplement: Supplementary file 8 [file Image10.TIF]

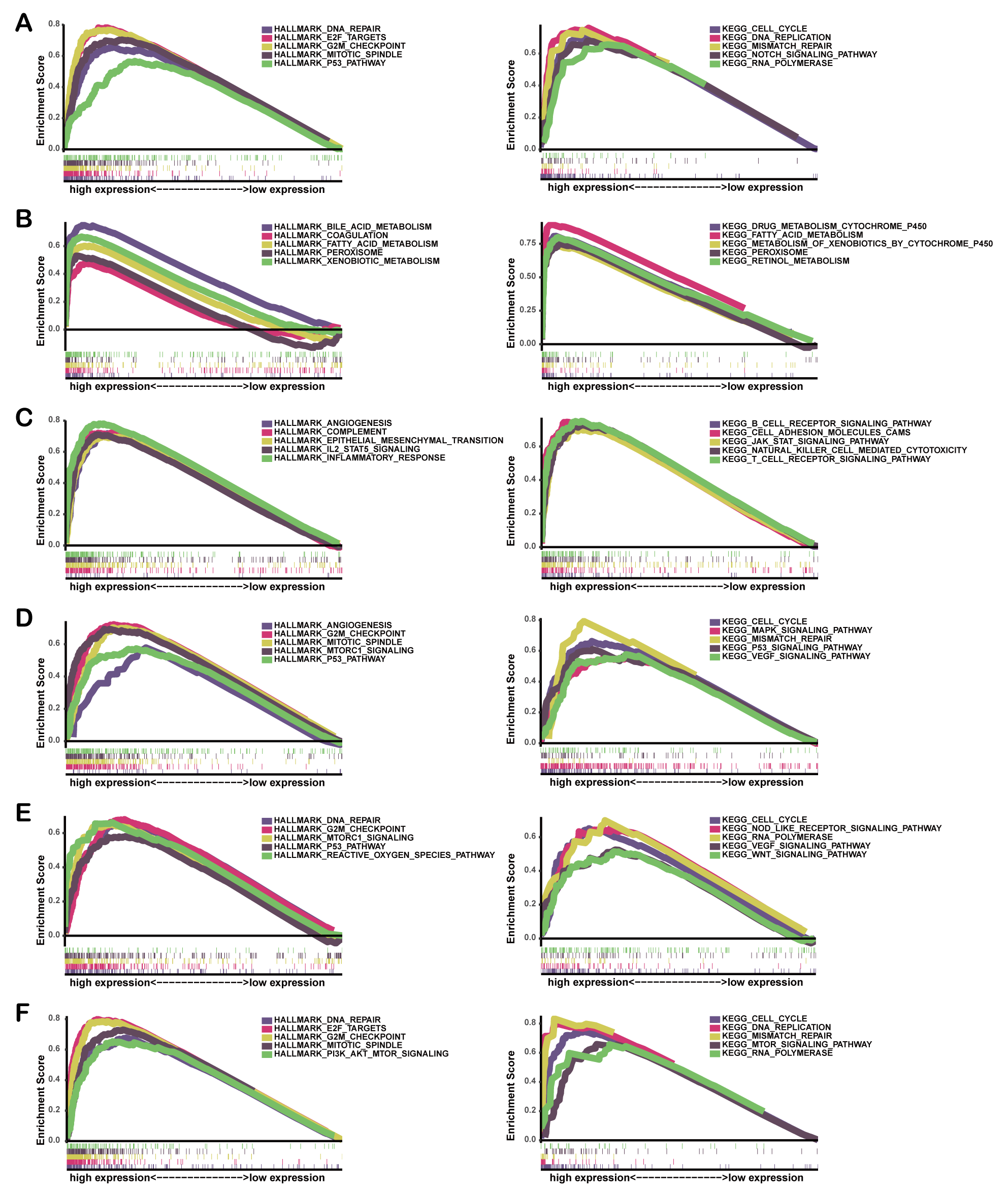

Supplement: Supplementary file 9 [file Image7.TIF]

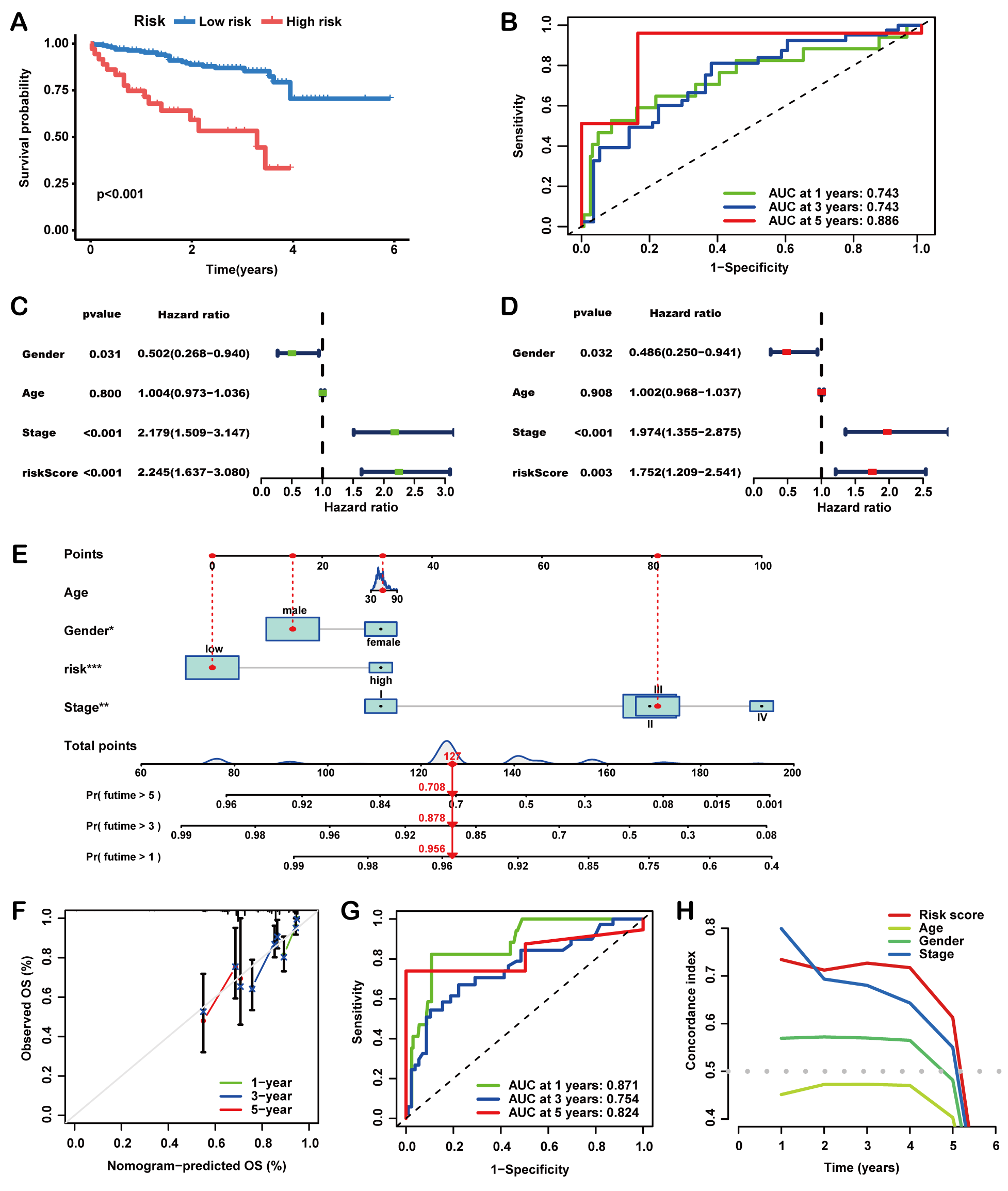

Supplement: Supplementary file 11 [file Image8.TIF]

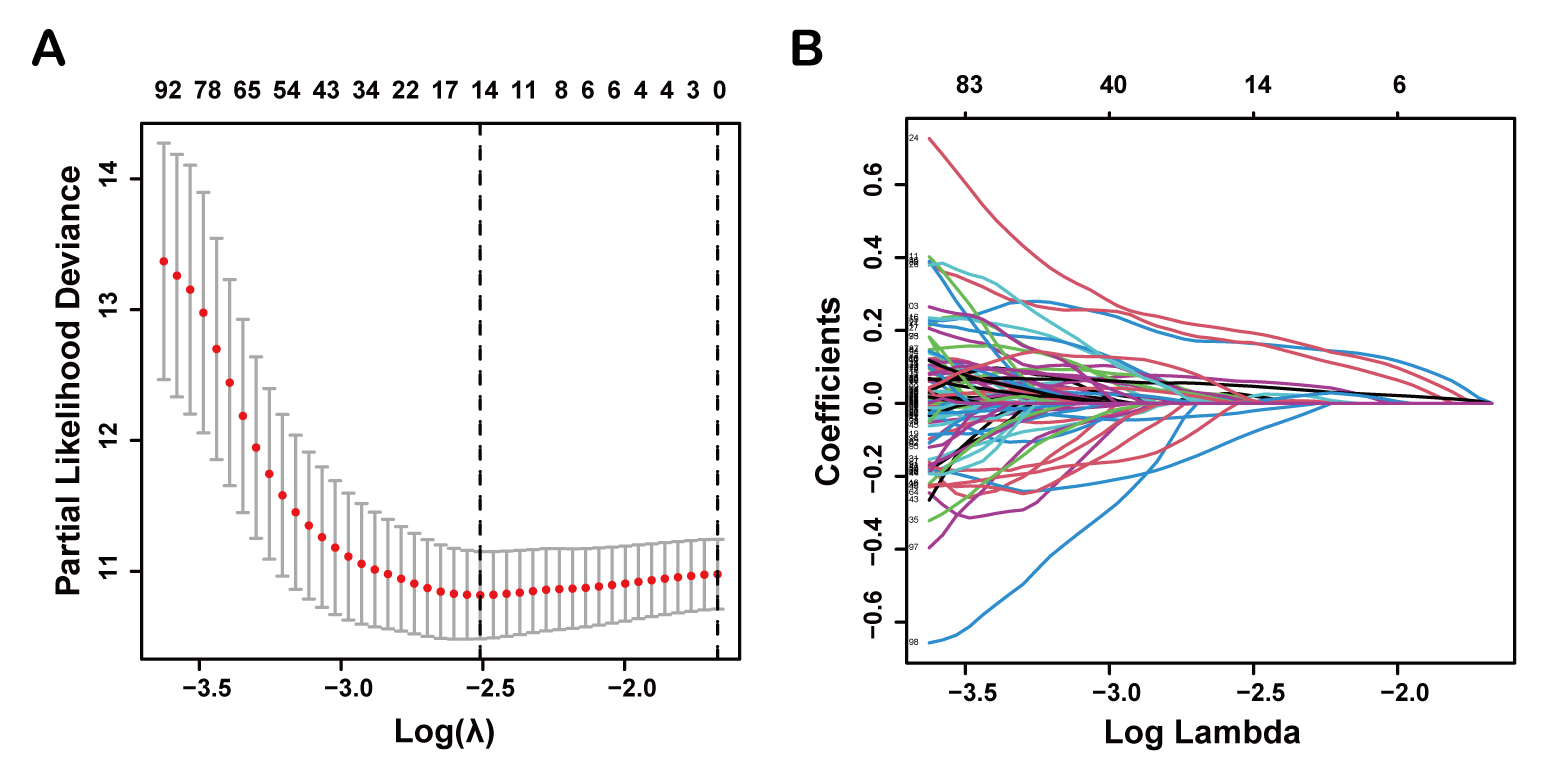

Supplement: Supplementary file 12 [file Image5.TIF]
