# Supplementary material for: Identifying a novel cuproptosis-related necroptosis gene subtype-related signature for predicting the prognosis, tumor microenvironment, and immunotherapy of hepatocellular carcinoma
Source: Front Mol Biosci. 2023 May 23;10:1165243. doi: 10.3389/fmolb.2023.1165243 (PMC10242026; doi:10.3389/fmolb.2023.1165243)
Supplement: Supplementary file 13 [file DataSheet1.docx]

## Supplementary Files

## Supplementary Figures


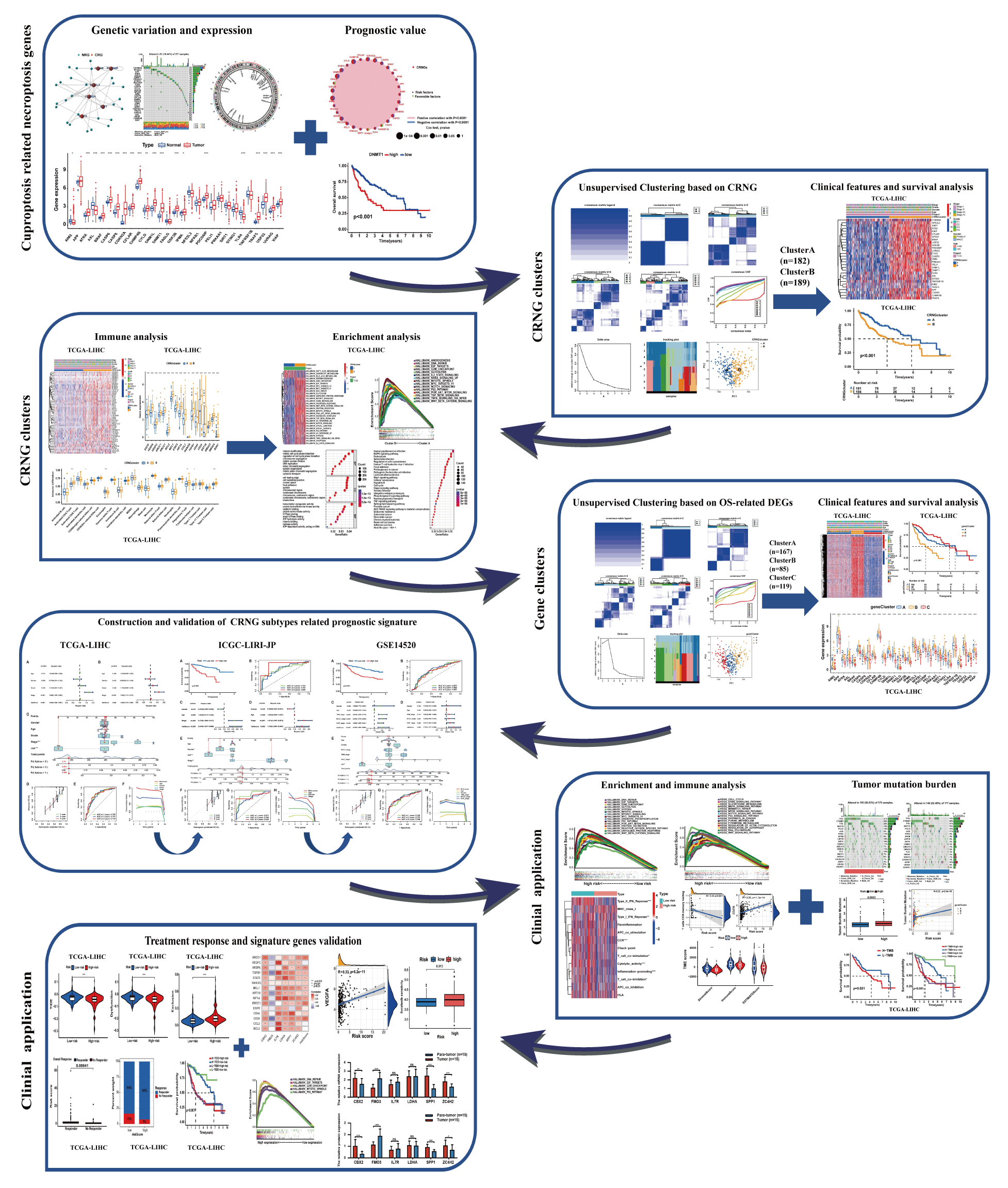


**Figure S1.** Flowchart of this work.


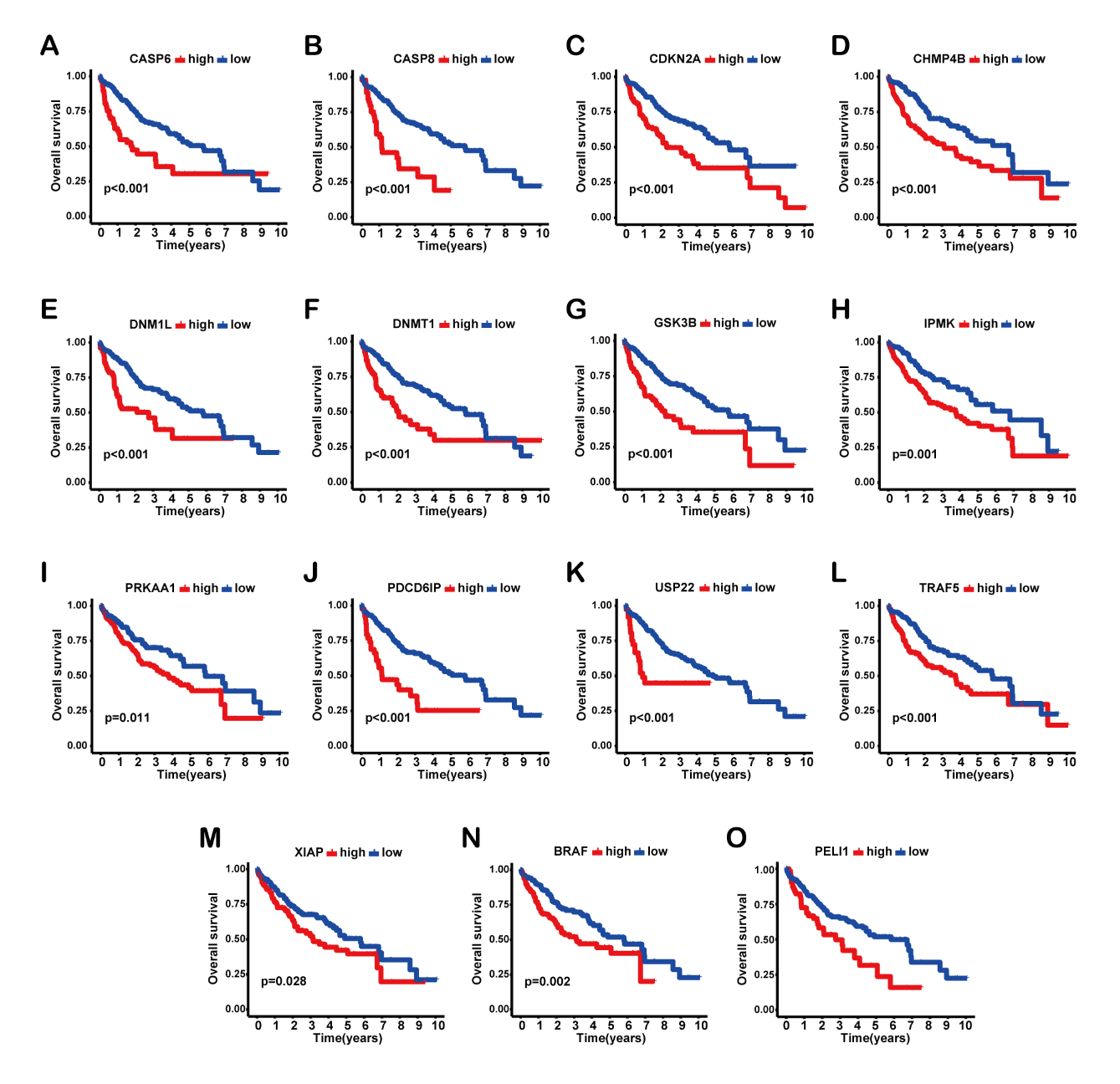


**Figure S2.** Kaplan-Meier survival curves of cuproptosis-related necroptosis genes in TCGA-LIHC cohort. (**A-O**) Kaplan-Meier survival curves between high and low expression levels of cuproptosis-related necroptosis genes.


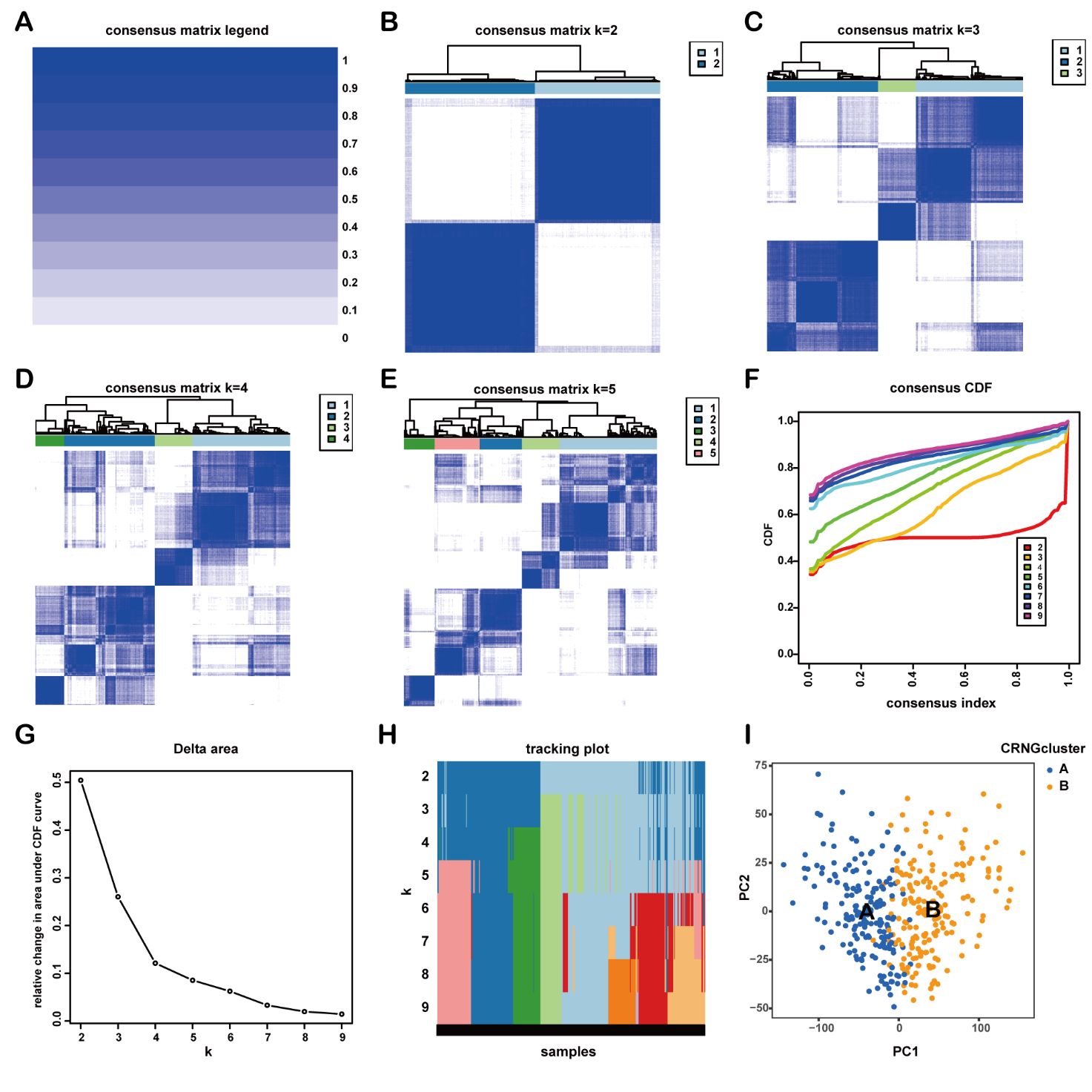


**Figure S3.** Unsupervised clustering for cuproptosis-related necroptosis genes(CRNGs). (**A-E**) Patients in TCGA-LIHC cohort was classified as 2 subtypes according to the consensus clustering matrix (k=2). (**F**) Uniform clustering CDF with k from 2 to 9. (**G**) Changes of the area under CDF curve with k from 2 to 9. (**H**) Tracking plot of the relationship between samples and clusters. (**I**) Principal component analysis (PCA) plot of the distribution of samples between 2 CRNG subtypes.


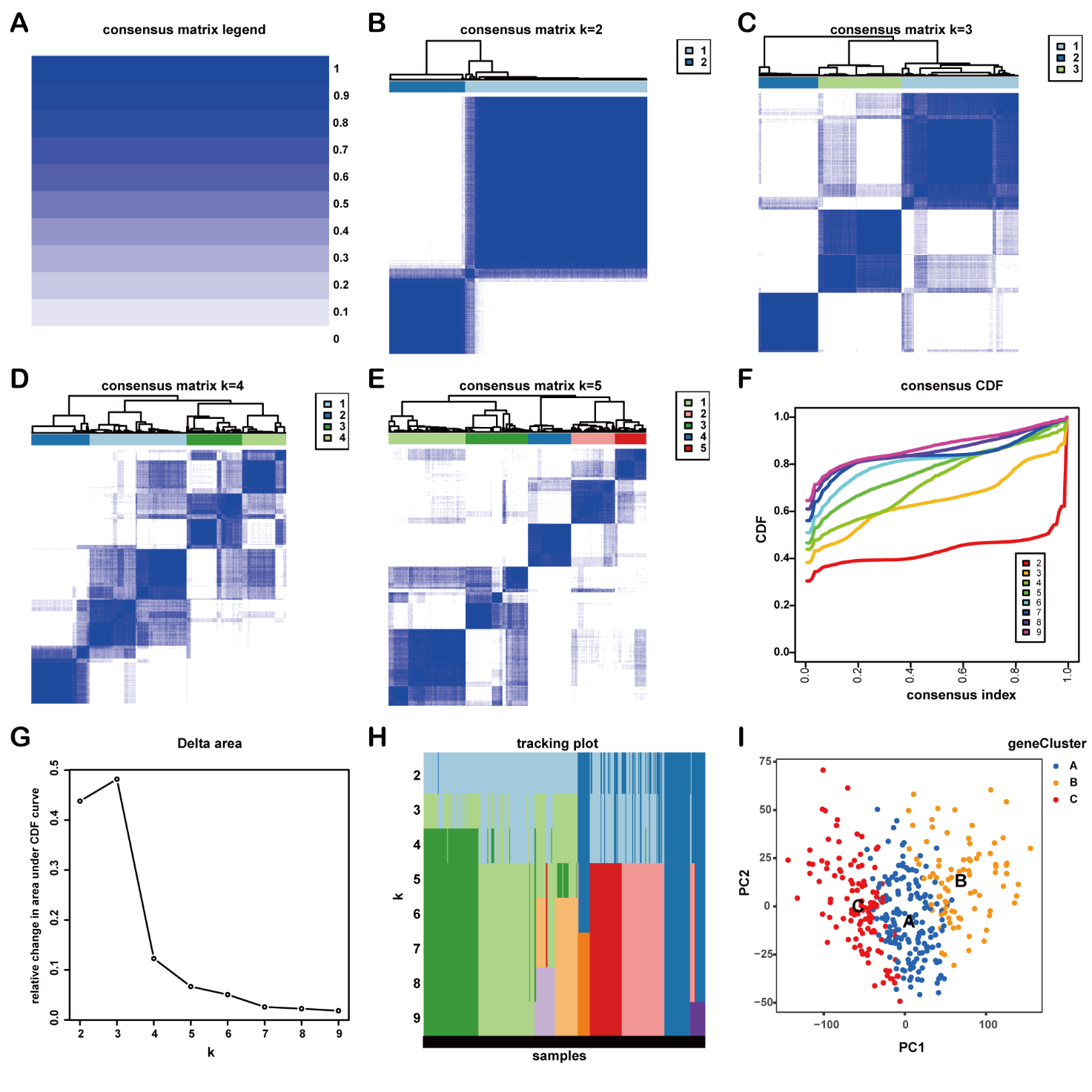


**Figure S4.** Unsupervised clustering for overall survival- differentially expressed genes(OS-DEGs) based on CRNGs. (**A-E**) Patients in TCGA-LIHC cohort was classified as 3 subtypes according to the consensus clustering matrix (k=3). (**F**) Uniform clustering CDF with k from 2 to 9. (**G**) Changes of the area under CDF curve with k from 2 to 9. (**H**) Tracking plot of the relationship between samples and clusters. (**I**) Principal component analysis (PCA) plot of the distribution of samples between 3 gene subtypes.


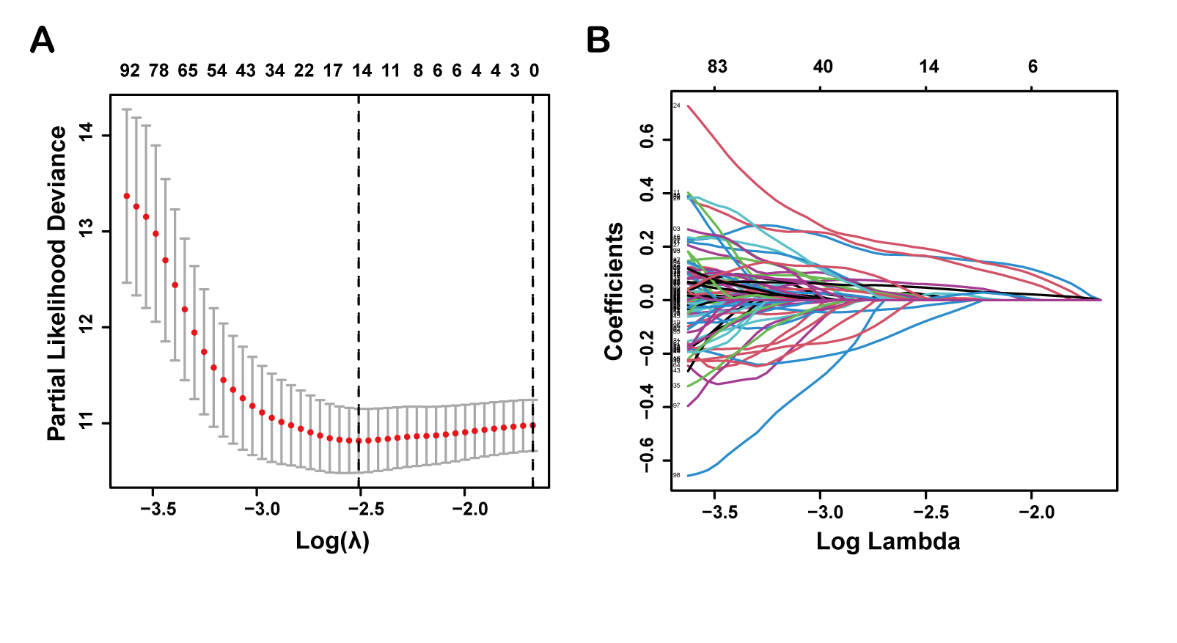


**Figure S5.** Lasso cox regression analysis. (**A**) LASSO coefficient profiles of overall survival- differentially expressed genes(OS-DEGs) based on CRNGs. (**B**) Cross-validation to confirm suitable tuning parameters in the LASSO regression. (LASSO, least absolute shrinkage and selection operator)


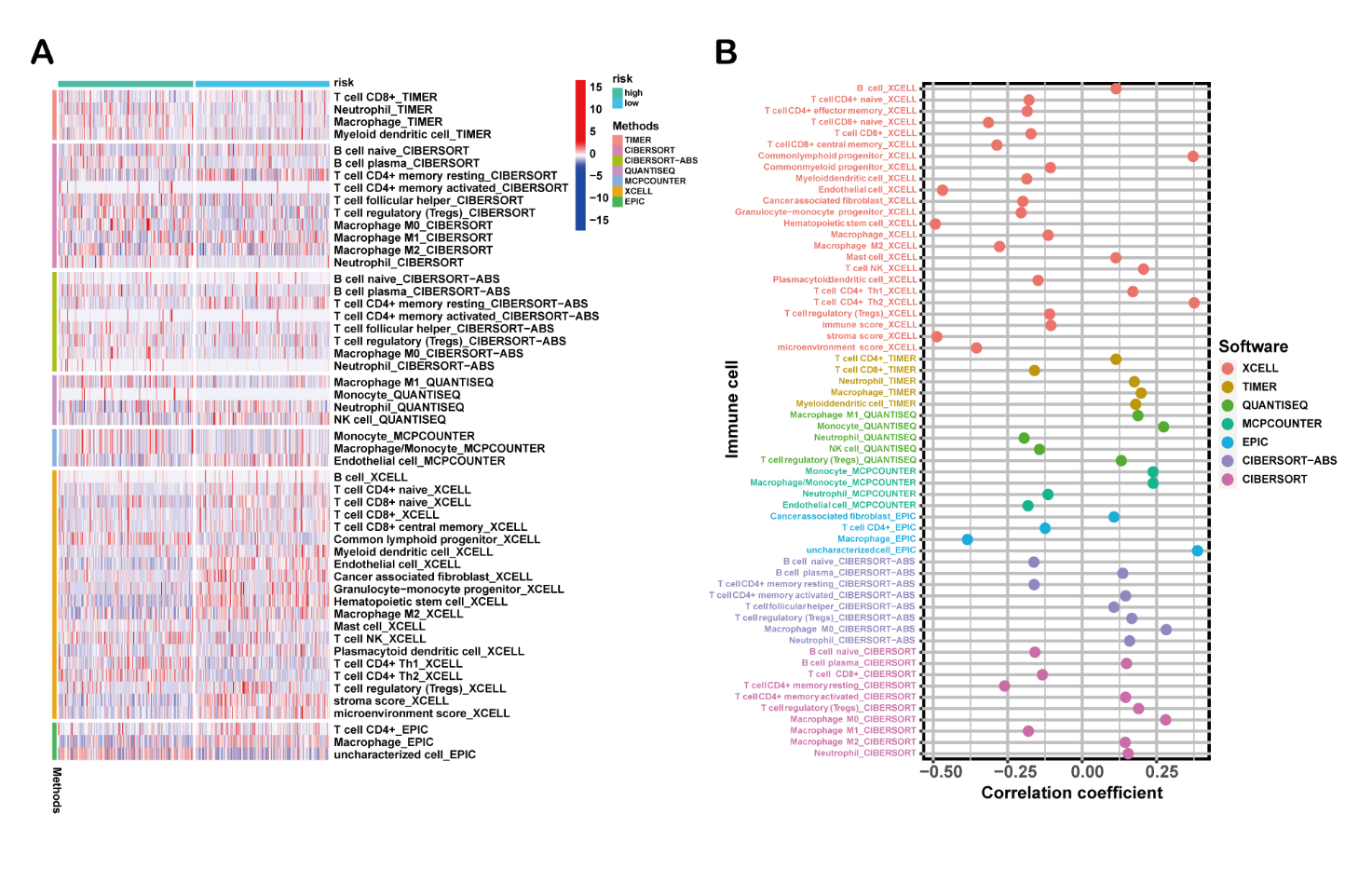


**Figure S6.** The correlation analysis between risk scores and immune cells infiltration with multiple immune infiltration algorithms. (**A**)The result of correlation analysis between risk scores and immune cells infiltration status with heatmap. (**B**) The result of correlation analysis between risk scores and immune cells infiltration status with lollipop plot.


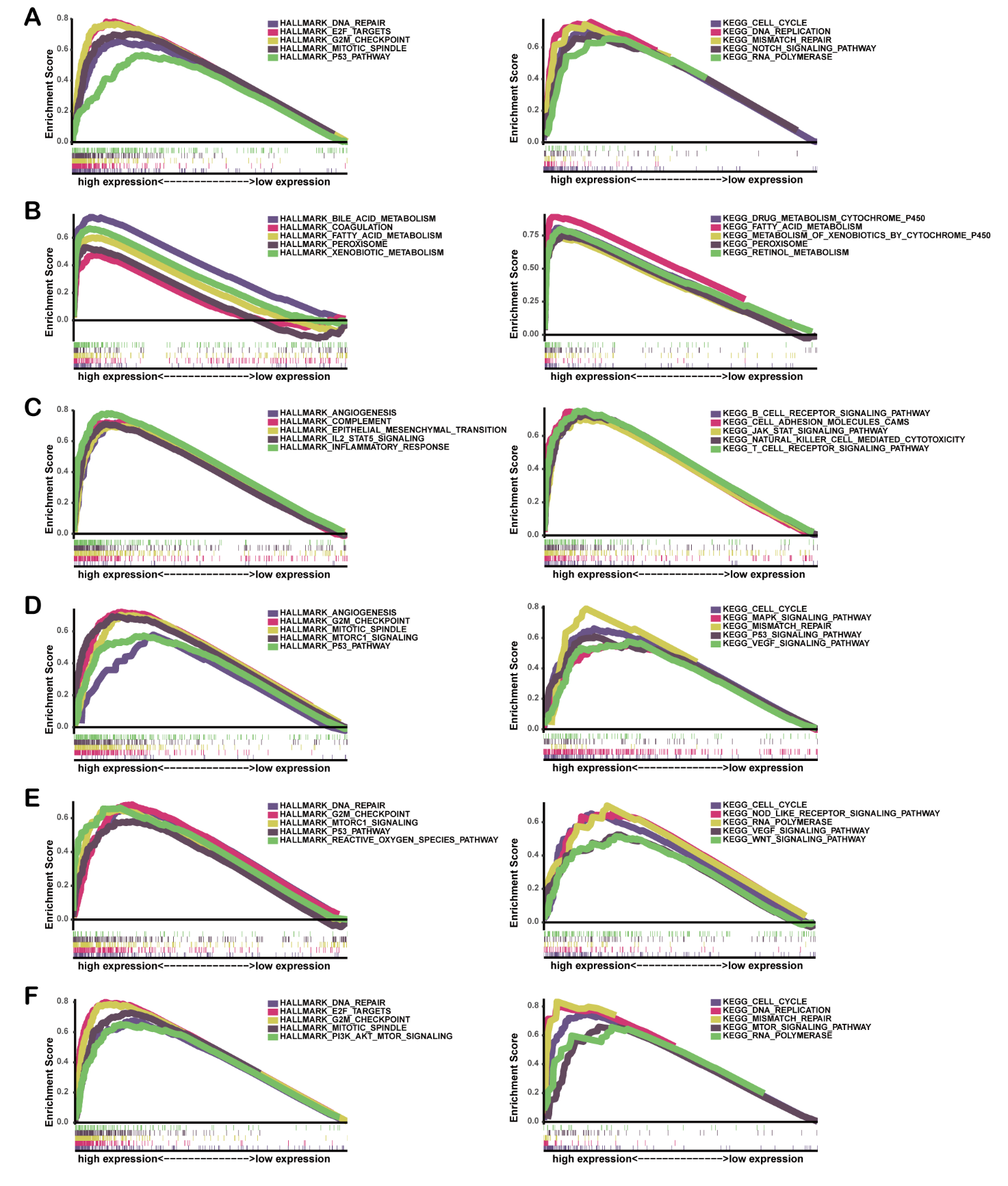


**Figure S7.** Gene set enrichment analysis (GSEA) of 6 genes in the prognostic model based on cuproptosis-related necroptosis genes. (**A**) GSEA of significant HALLMARK and KEGG terms between high and low expression levels of CBX2. (**B**) GSEA of significant HALLMARK and KEGG terms between high and low expression levels of FMO3. (**C**) GSEA of significant HALLMARK and KEGG terms between high and low expression levels of IL7R. (**D**) GSEA of significant HALLMARK and KEGG terms between high and low expression levels of LDHA. (**E**) GSEA of significant HALLMARK and KEGG terms between high and low expression levels of SPP1. (**F**) GSEA of significant HALLMARK and KEGG terms between high and low expression levels of ZC4H2.


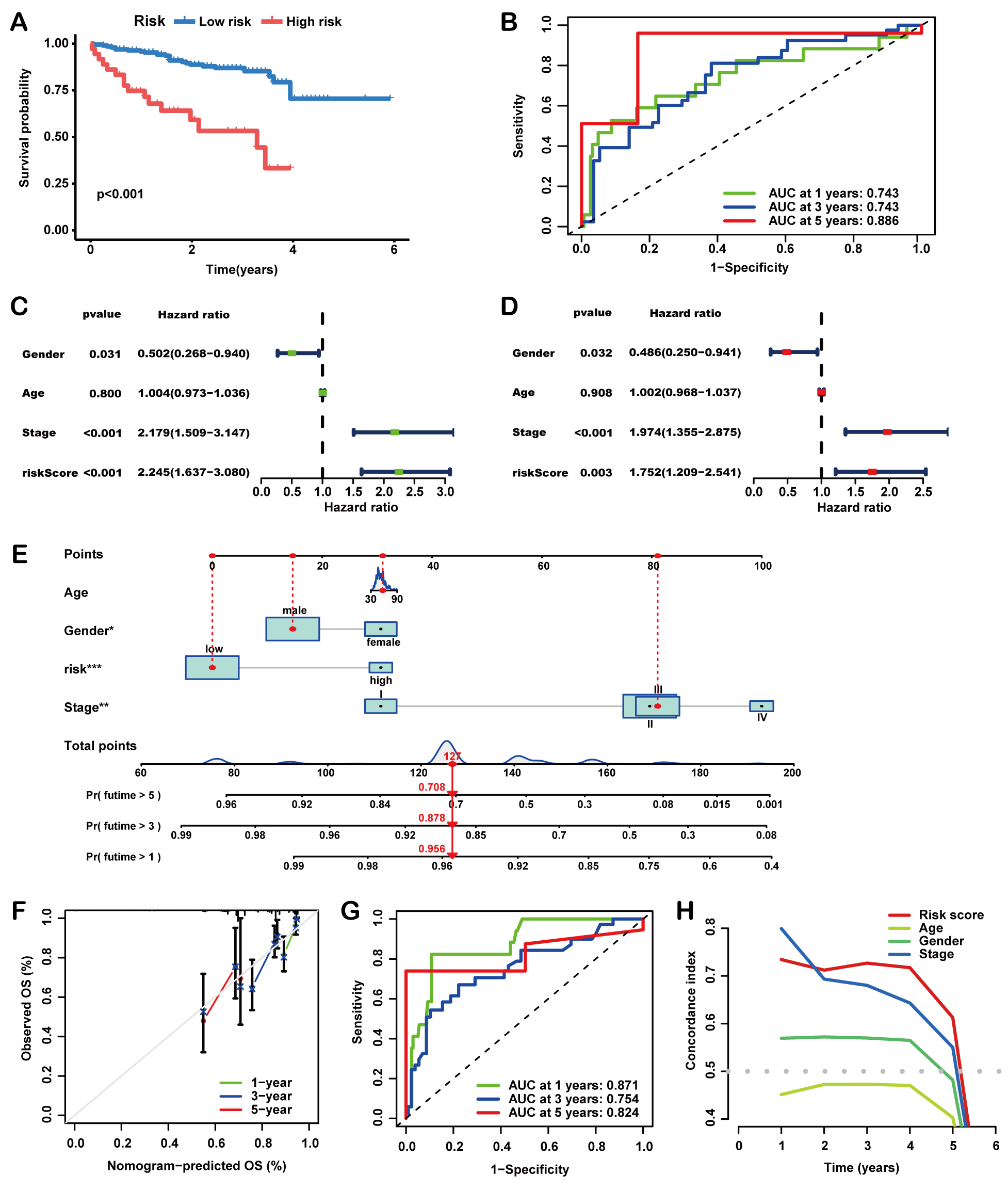


**Figure S8.** Validation of prognostic signature in ICGC-LIRI-JP cohort. (**A**) Survival analysis on patients with different risk profiles. (**B**) ROC analysis on patients with different risk profiles. (**C**)Analysis of cox regression with univariate variables on prognostic signature and clinical characteristics of patients in ICGC- LIRI-JP cohort. (**D**) Analysis of cox regression with multivariate variables on prognostic signature and clinical characteristics of patients in ICGC-LIRI-JP cohort. (**E**) Construction of a nomogram for predicting OS probability of patients in ICGC-LIRI-JP cohort. (**F**) Assessment for predictive value of the nomogram for patients in ICGC-LIRI-JP cohort with calibration curves. (**G**) Assessment for predictive value of the nomogram for patients in ICGC-LIRI-JP cohort with ROC curves. (**H**) C-index analysis for independent prognostic value of the signature in ICGC-LIRI-JP cohort.


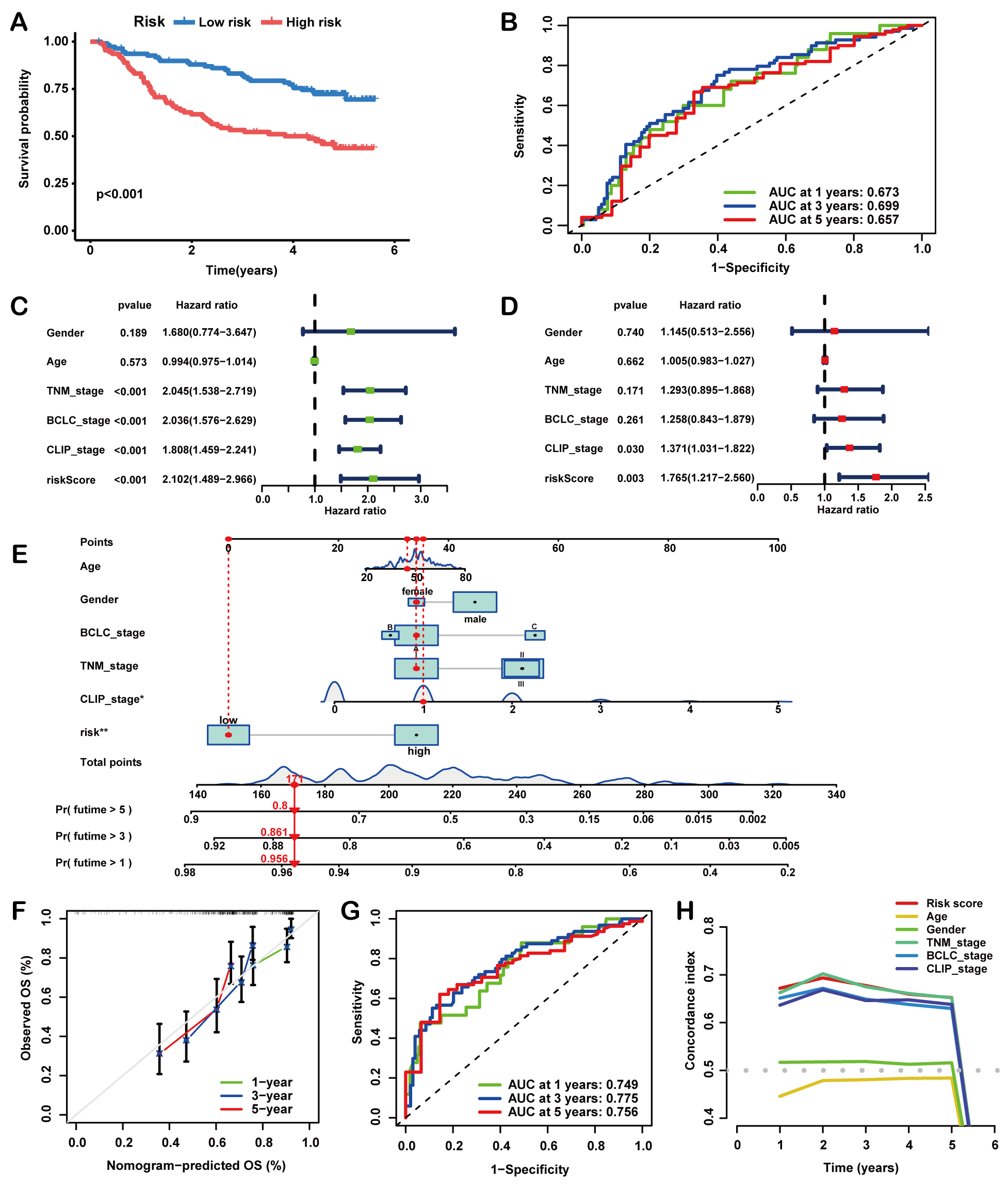


**Figure S9.** Validation of prognostic signature in GSE14520 cohort. (**A**) Survival analysis on patients with different risk profiles. (**B**) ROC analysis on patients with different risk profiles. (**C**)Analysis of cox regression with univariate variables on prognostic signature and clinical characteristics of patients in GSE14520 cohort. (**D**) Analysis of cox regression with multivariate variables on prognostic signature and clinical characteristics of patients in GSE14520 cohort. (**E**) Construction of a nomogram for predicting OS probability of patients in GSE14520 cohort. (**F**) Assessment for predictive value of the nomogram for patients in GSE14520 cohort with calibration curves. (**G**) Assessment for predictive value of the nomogram for patients in GSE14520 cohort with ROC curves. (**H**) C-index analysis for independent prognostic value of the signature in GSE14520cohort.


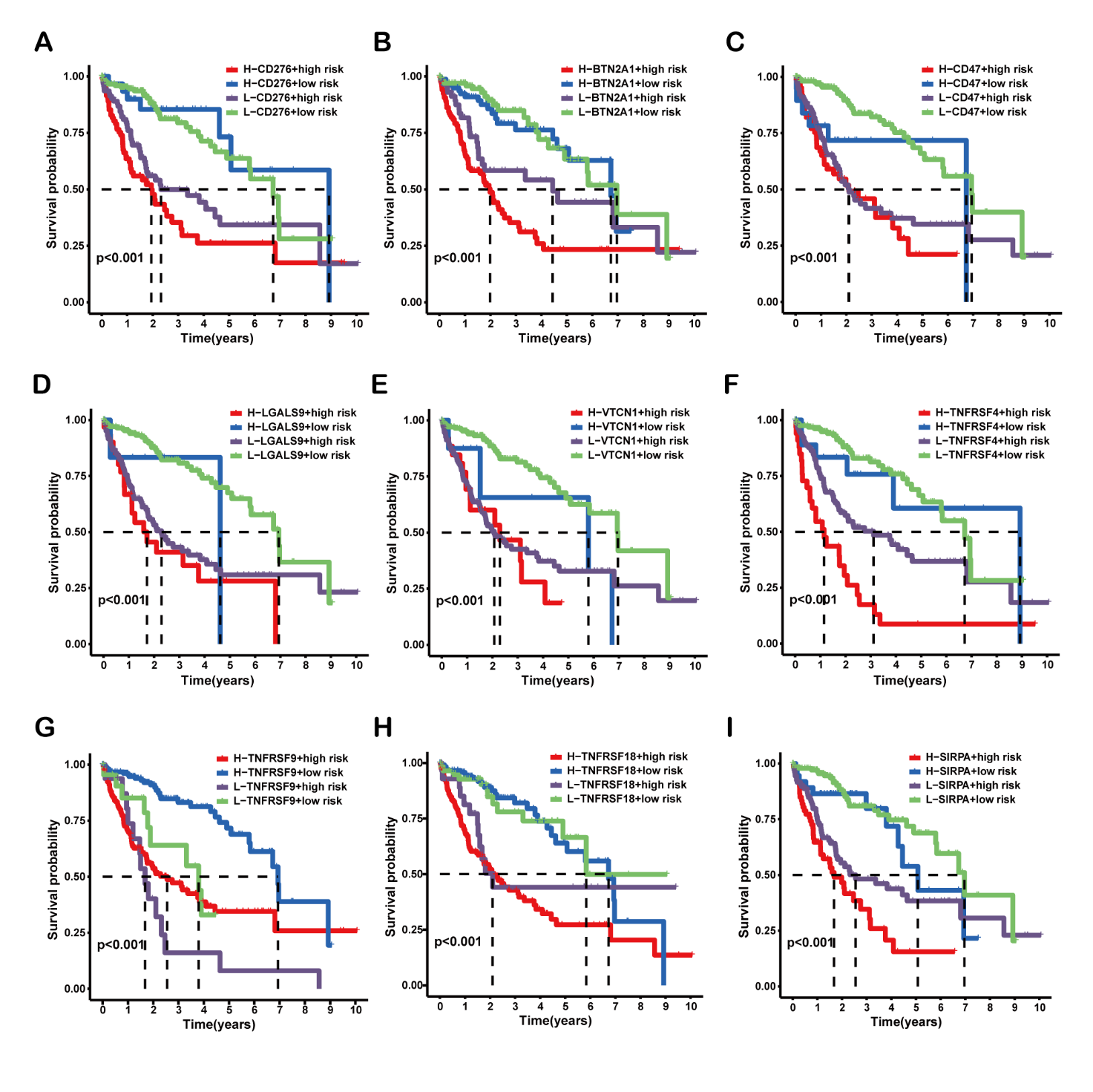


**Figure S10.** The Kaplan–Meier survival curves among 4 groups classified by risk-group and the expression levels of immune checkpoint genes (ICGs) in TCGA-LIHC cohort. (**A-I**) The Kaplan–Meier survival curves among 4 groups classified by risk-group and the expression levels of ICGs.

## Supplementary Tables

Table S1. Summary of 19 cuproptosis related genes.

Table S2. Summary of 140 necroptosis related genes.

Table S3. Identification of cuproptosis related necroptosis genes.

Table S4. Summary of 29 cuproptosis related necroptosis genes.

Table S5. Clinicopathological information of HCC patients used in this study.

Table S6. Information of primers sequences and antibodies involved in this study.

Table S7. Univariate Cox regression analysis of 29 cuproptosis related necroptosis genes in HCC patients.

Table S8. Multivariate Cox regression analysis of 15 cuproptosis related necroptosis genes associated with overall survival in HCC patients.

Table S9. The activation states of HALLMARK and KEGG terms between CRNG clusters by GSVA.

Table S10. The activation states of HALLMARK and KEGG terms between CRNG clusters by GSEA.

Table S11. Functional annotation of DEGs between two CNRG clusters.

Table S12. GSEA of significant HALLMARK and KEGG terms between CBX2 high- and low-expression group.

Table S13. GSEA of significant HALLMARK and KEGG terms between FMO3 high- and low-expression group.

Table S14. GSEA of significant HALLMARK and KEGG terms between IL7R high- and low-expression group.

Table S15. GSEA of significant HALLMARK and KEGG terms between LDHA high- and low-expression group.

Table S16. GSEA of significant HALLMARK and KEGG terms between SPP1 high- and low-expression group.

Table S17. GSEA of significant HALLMARK and KEGG terms between ZC4H2 high- and low-expression group.

Table S18. Clinicopathological information of patients in ICGC-LIRI-JP cohort and GSE14520 cohort.

Table S19. GSEA of significant HALLMARK and KEGG terms between high- and low-risk group.

Table S20. Correlations between the expression of ICGs and risk score.

Table S21. Correlations between the expression of chemotherapy resistance related genes and risk score.
